# Morphometric analysis of fossil bumble bees (Hymenoptera, Apidae, Bombini) reveals their taxonomic affinities

**DOI:** 10.3897/zookeys.891.36027

**Published:** 2019-11-21

**Authors:** Manuel Dehon, Michael S. Engel, Maxence Gérard, A. Murat Aytekin, Guillaume Ghisbain, Paul H. Williams, Pierre Rasmont, Denis Michez

**Affiliations:** 1 Laboratory of Zoology, Research Institute of Biosciences, University of Mons, Place du parc 20, 7000 Mons, Belgium; 2 Division of Invertebrate Zoology, American Museum of Natural History, Central Park West at 79th, New York, NY 10024-5192, USA; 3 Division of Entomology, Natural History Museum, and Department of Ecology and Evolutionary Biology, University of Kansas, 1501 Crestline Drive – Suite 140, Lawrence, KS 66045, USA; 4 Pamukkale Sitesi, B Blok, Çayyolu, Ankara, Turkey; 5 Department of Life Sciences, Natural History Museum, Cromwell Road, London SW7 5BD, UK

**Keywords:** *
Bombus
*, evolution, fossil, geometric morphometrics, review, taxonomy

## Abstract

Bumble bees (*Bombus* spp.) are a widespread corbiculate lineage (Apinae: Corbiculata: Bombini), mostly found among temperate and alpine ecosystems. Approximately 260 species have been recognized and grouped recently into a simplified system of 15 subgenera. Most of the species are nest-building and primitively eusocial. Species of *Bombus* have been more intensely studied than any other lineages of bees with the exception of the honey bees. However, most bumble bee fossils are poorly described and documented, making their placement relative to other *Bombus* uncertain. A large portion of the known and presumed bumble bee fossils were re-examined in an attempt to better understand their affinities with extant Bombini. The taxonomic affinities of fossil specimens were re-assessed based on morphological features and previous descriptions, and for 13 specimens based on geometric morphometrics of forewing shape. None of the specimens coming from Eocene and Oligocene deposits were assigned within the contemporary shape space of any subgenus of *Bombus*. It is shown that *Calyptapis florissantensis* Cockerell, 1906 (Eocene-Oligocene boundary, Florissant shale, Colorado, USA) and *Oligobombus cuspidatus* Antropov, 2014 (Late Eocene, Bembridge Marls) likely belong to stem-group Bombini. *Bombus anacolus* Zhang, 1994, *B. dilectus* Zhang, 1994, *B. luianus* Zhang, 1990 (Middle Miocene, Shanwang Formation), as well as *B. vetustus* Rasnitsyn & Michener, 1991 (Miocene, Botchi Formation) are considered as species inquirenda. In the Miocene, affinities of fossils with derived subgenera of *Bombus* s. l. increased, and some are included in the shape space of contemporary subgenera: *Cullumanobombus* (i.e., *B. pristinus* Unger, 1867, *B. randeckensis* Wappler & Engel, 2012, and *B. trophonius* Prokop, Dehon, Michez & Engel, 2017), *Melanobombus* (i.e., *B. cerdanyensis* Dehon, De Meulemeester & Engel, 2014), and *Mendacibombus* (i.e., *B. beskonakensis* (Nel & Petrulevičius, 2003), new combination), agreeing with previous estimates of diversification.

## Introduction

Bumble bees (Bombini: *Bombus* Latreille, 1802) are a lineage of corbiculate bees (Apidae: Apinae) dominant in many temperate and alpine ecosystems (Williams 1998; Michener 2007). Like almost all bees, they feed entirely on pollen for protein and lipid resources, and nectar for carbohydrates. *Bombus* are valuable for agricultural pollination (e.g., Pouvreau 1984; Plowright and Laverty 1987), and have been domesticated since the 1970s (Röseler 1973), resulting in commercial rearing with probably several millions of colonies produced per year (Velthuis and Doorn 2006; Goulson 2010). Approximately 260 species have been recognized (Williams 1998) and grouped into a simplified system of 15 subgenera (Williams et al. 2008). Most of the species are nest-building (i.e., females collect pollen using a corbicula) and primitively eusocial, meaning that the life cycle includes a solitary queen stage (Heinrich 1979). However, several species are social parasites: all species of the subgenus Psithyrus Lepeletier, 1832, and the species Bombus (Thoracobombus) inexspectatus (Tkalců, 1963), B. (Alpinobombus) hyperboreus Schoenherr, 1809, and B. (Alpinobombus) natvigi Richards 1931 (Hines and Cameron 2010; Brasero et al. 2018; Williams et al. 2019). Morphologically, the genus *Bombus* is characterized by an intermediate to very large body size (9–22 mm long), often with conspicuous color patterns (Williams 2007), the presence of outer mandibular grooves, an apically closed forewing marginal cell, the presence of an auricle at the metatibia-metabasitarsus junction, the presence of a supra-alar carina, the hamuli not reduced on the hind wing margin, the absence of a jugal lobe, and glabrous compound eyes (Engel 2001; Michener 1990, 2007; Engel and Rasmussen in press). In females, the pretarsal claws are cleft, with a small arolia present, and the metatibial spurs are present (Engel 2001; Michener 1990, 2007; Engel and Rasmussen in press). Both wings have strong and complete venation (Michener 1990, 2007). In the forewing, the marginal cell is longer than the distance from its apex to the forewing tip; the pterostigma is small, scarcely longer than the prestigma; r-rs arises near or beyond the middle of the pterostigma; and the margin within the marginal cell is straight or commonly concave. *Bombus* s. l. display interspecific diversity in structures like male genitalia, female sting, color pattern, and mandibular shape (Engel 2001; Michener 1990, 2007).

Bumble bees have been more intensely studied than other lineages of bees with the exception of the honey bees (Apini: *Apis* L., 1758) (Michener 2007). Those studies include taxonomic and cladistic investigations, many of them focusing on the recovery of a robust hypothesis of phylogenetic relationships among and within the subgenera (e.g., Vogt 1911; Milliron 1961, 1973a, b; Tkalců 1972; Pekkarinen 1979; Pekkarinen et al. 1979; Obrecht and Scholl 1981; Ito 1985; Williams 1985; Pamilo et al. 1987; Cameron et al. 2007). Bumble bees exhibit higher species diversity in cooler climates of the Holarctic region, with more species and subgenera in Eurasia than in North America (Williams 1998). Historical patterns of dispersal among the continents and climatic associations of bumble bee origins were described by Skorikov (1923), Williams (1985), Kawakita et al. (2004), and Williams et al. (2018). Hines (2008) recently estimated divergence times using fossil calibrations and molecular rates derived from the literature. However, she purposefully excluded fossils of *Bombus* s. l. for her analyses, and instead considered that reliable bumble bee fossils were too poorly preserved to reveal good morphological synapomorphies for placement within *Bombus* s. l. Hines (2008) therefore decided to use outgroup fossils and subfossils as calibration points [i.e., the subfossil stingless bees *Liotrigona vetula* Moure & Camargo, 1978, *Hypotrigona gribodoi* (Magretti, 1884), and fossil meliponines *Liotrigonopsis rozeni* Engel, 2001, *Kelneriapis eocenica* (Kelner-Pillault, 1969), and *Proplebeia dominicana* (Wille & Chandler, 1964)]. Those analyses estimated that the crown group of extant *Bombus* s. l. originated in the Upper Eocene to Middle Oligocene, i.e., 40.0–25.0 Ma, perhaps near the Eocene-Oligocene boundary (i.e., 34.0 Ma). It is unclear whether the purposeful exclusion of *Cretotrigona prisca* (Michener & Grimaldi, 1988), from 70 Ma, as an outgroup calibration point impacted the overall estimated divergence times obtained by Hines (2008). Regardless, the Eocene-Oligocene transition is a well-documented global cooling period that resulted in significant extinctions, particularly across the Northern Hemisphere (Zachos et al. 2008; Hansen et al. 2013). An Old World ancestor of extant *Bombus* s. l. was supported, with early dispersal events from the Old World into the New World and North America to South America (Williams 1985; Hines 2008). In the phylogenetic tree presented in Cameron et al. (2007) and Hines (2008), *Mendacibombus* is sister to all other clades, while extant species of this subgenus are estimated to have diverged in the last 10 Ma (Williams et al. 2016). A global revision of available bumble bee fossils is needed to corroborate or reject temporal hypotheses proposed by Hines (2008), and more critically the discovery of more and better-preserved fossil bombines is needed as the record of this interesting tribe is quite scant.

*Bombus* is the only contemporary genus of the tribe Bombini but additional fossils have been associated with this tribe, and these have either been proposed within the genus, or in putatively extinct genera. Overall, the fossil record of bees is comparatively scarce, with only around 200 described species (e.g., Kotthoff et al. 2011; Michez et al. 2012; Wappler et al. 2012; Engel and Michener 2013a; Engel and Breitkreuz 2013; Engel et al. 2013, 2014, 2018; Dewulf et al. 2014; Dehon et al. 2014, 2017; Engel 2014, 2019a, b; Prokop et al. 2017). In total, 14 bombine fossil species have been described, each described from a single specimen with the exception of *Calyptapis florissantensis* Cockerell which was documented from two specimens (Table 1). Most of these are poorly described and documented, making their placement relative to extant *Bombus* uncertain. The majority of the specimens were found in Miocene sediments and have been described in the genera *Bombus* Latreille, 1802, *Oligoapis* Nel & Petrulevičius, 2003, *Oligobombus* Antropov, 2014, *Paraelectrobombus* Nel & Petrulevičius, 2003, and *Calyptapis* Cockerell, 1906 (Table 1). The aim of the present study is to provide a taxonomic overview of the available fossil bumble bees and to evaluate their affinities with extant taxa. Using landmark-based geometric morphometric analyses of the forewing shape and morphology, we estimate the similarity/dissimilarity of the fossil wing shape with extant and extinct bee taxa, particularly other corbiculate bees (Apinae: Corbiculata). Based on the results of the forewing shape comparisons, we propose a new taxonomic arrangement for many of the fossils. Based on our revised system we re-examined whether these few occurrences have any impact on understanding the diversification and extinction patterns of bumble bees.

**Table 1.** All known fossils described as bumble bees (genus *Bombus*) or as closely allied genera. Linear Discriminant Analysis (LDA) 1–3 are based on dataset 1. LDA 4 is based on dataset 2. LDA 5 is based on dataset 3. Key: * = fossil specimen without forewing picture/drawing available for geometric morphometric analyses. Abbreviations: Ap. = Apidae, B = Bombini. E = Electrapini. H = Holotype. S2: second specimen described by Cockerell (1908). *B.* = *Bombus*. *C.* = *Calyptapis*. *O.* = *Oligoapis*. *Ol.* = *Oligobombus*. *P. = Paraelectrobombus*. ° = the specimen was included in the shape space of the most similar clade. a = Jiménez-Moreno et al. (2010). b = Bachmayer et al. (1971). c = Akhmetjev (1973). d = Yang et al. (2007). e = Heizmann (1983). f = J. Prokop, pers. comm. (Wappler et al. 2012). g = Knor et al. (2012). h = Gray and Kittleman (1967). i = Paichelier et al. (1978). j = Evanoff et al. (2001). k = Antropov et al. (2014).

Taxon	Reference	Age (Ma)	Locality	LDA1	LDA2	LDA3	LDA4	LDA5	New taxonomic hypothesis
*B. cerdanyensis*	Dehon et al. (2014)	10.0*^a^*	La Cerdanya ES	Ap.	Apinae	Bombini	Bombini°	*Melanobombus*°	B. (Melanobombus) cerdanyensis
B. ? pristinus	Unger (1867)	11.2–7.1*^b^*	Euboea GR	Ap.	Apinae	Bombini	Bombini°	*Cullumanobombus*°	B. (Cullumanobombus) pristinus
*B. vetustus*	Rasnitsyn and Michener (1991)	11.2–7.1*^c^*	Botchi River RU	Ap.	Eucerinae	Bombini	Bombini	* Bombias *	*B. vetustus* sp. inq.
*B. anacolus*	Zhang et al. (1994)	17.0–15.2*^d^*	Shandong CN	Ap.	Apinae	Bombini	Bombini	* Mendacibombus *	*B. anacolus* sp. inq.
*B. dilectus*	Zhang et al. (1994)	17.0–15.2*^d^*	Shandong CN	Ap.	Apinae	Tetrapediini	Bombini	* Bombias *	*B. dilectus* sp. inq.
*B. luianus*	Zhang (1990)	17.0–15.2*^d^*	Shandong CN	Ap.	Apinae	Bombini	Bombini°	* Melanobombus *	*B. luianus* sp. inq.
*B. randeckensis*	Wappler et al. (2012)	18.0–16.0*^e^*	Randeck Maar DE	Ap.	Apinae	Bombini	Bombini°	*Cullumanobombus*°	B. (Cullumanobombus) randeckensis
B. ? crassipes*	Novák (1877)	18.0–17.0*^f^*	Krottensee CZ	–	–	–	–	–	*B. crassipes*
*B. trophonius*	Prokop et al. (2003)	20.0*^g^*	Bilina Mine CZ	Ap.	Apinae	Bombini	Bombini°	*Cullumanobobmus*°	B. (Cullumanobombus) trophonius
B. proavus*	Cockerell (1931)	21.3–12.1*^h^*	Latah US	–	–	–	–	–	*B. proavus*
*O. beskonakensis*	Nel and Petrulevičius (2003)	22.5*^i^*	Bes-Konak TR	Ap.	Apinae	Bombini	Bombini°	* Mendacibombus *	B. (Mendacibombus) beskonakensis comb.n.
*P. patriciae*	Nel and Petrulevičius (2003)	22.5*^i^*	Bes-Konak TR	Ap.	Apinae	Bombini	Bombini	* Mendacibombus *	B. (Paraelectrobombus) patriciae comb.n.
*C. florissantensis*	Cockerell (1906)	37.0–33.9*^j^*	Florissant shale US	Ap.	H: Apinae; S2: Eucerinae	Electrapini	H: B; S2: E	* Bombias *	*C. florissantensis*
*Ol. cusipdatus*	Antropov et al. (2014)	36.0^k^	Isle of Wight UK	Ap.	Apinae	Electrapini	Bombini	* Bombias *	*Ol. cuspidatus*

## Materials and methods

### Type revision, morphological terminology, and classification

**Figure 1.**
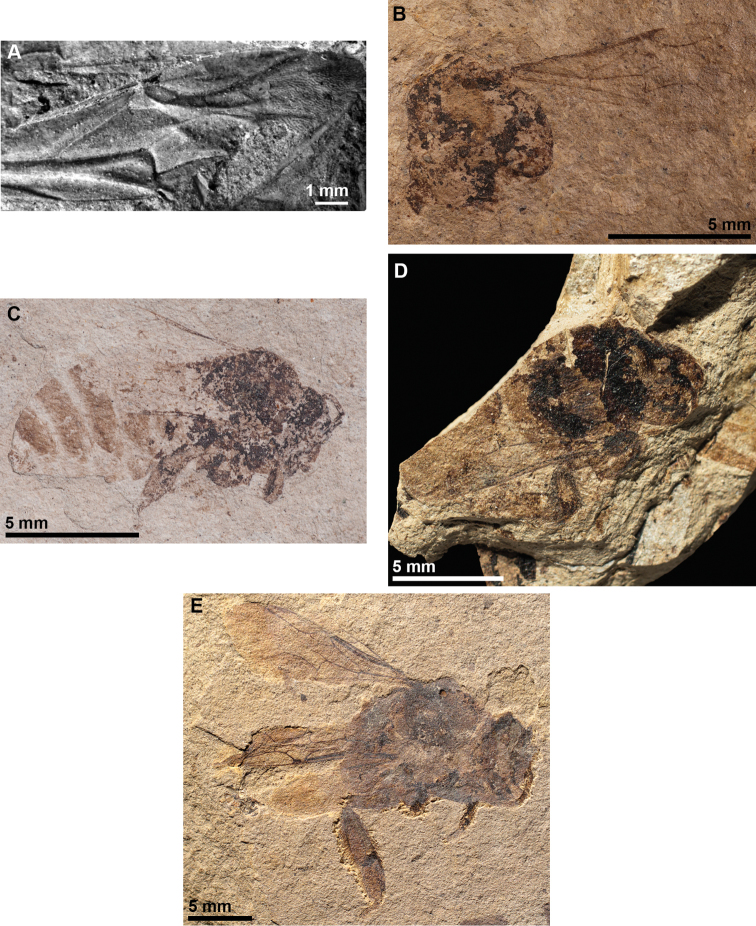
Representative fossil bumble bees **A***Oligobombus cuspidatus* (photograph by Antropov et al. (2014)) **B** Holotype of *Calyptapis florissantensis* (photograph by Manuel Dehon) **C***C. florissantensis* (photograph by Talia S. Karim) **D**Bombus (Paraelectrobombus) patriciae (photograph by Gaëlle Doitteau) **E**B. (Mendacibombus) beskonakensis (photograph by Gaëlle Doitteau).

We examined all of the fossils described in the literature as bumble bees or as closely allied extinct genera (Table 1), corresponding to 15 specimens representing 14 described species. For all species, we tried to locate the type material to check against the original description and to better illustrate the fossil, if needed. We contacted the potential repositories of the fossils and were able to locate 13 specimens for review (Fig. 1). Information about museum repositories is included in the “Results” section. Overall, we gathered pictures and/or drawings of the forewings of 13 specimens representing 12 of 14 species (Table 1).

The morphological terminology follows that of Engel (2001) and Michener (2007), while the higher classification (i.e., subfamily, tribe) follows that of Michener (2007) (i.e., seven families: Andrenidae, Apidae, Colletidae, Halictidae, Megachilidae, Melittidae, and Stenotritidae). For bumble bees, we used the subgeneric system of Williams et al. (2008) where 15 subgenera were proposed. A complete list of extant species with their nomenclature is available at the following link (updated from Williams 1998): (http://www.nhm.ac.uk/research-curation/research/projects/bombus/groups.html).

### Geological settings

Fossils of bumble bees have been described from eleven deposits from the Late Eocene to the Upper Miocene: Brembridge Marls, Florissant, BesKonak, Latah, Bílina Mine, Krottensee, Randeck Maar, Shandong, Botchi River, Euboea, and La Cerdanya (Table 1).

The Insect Bed of the Bembridge Marls from the Late Eocene (i.e., 36.0 Ma) is located on the Isle of Wight (UK). Two bee fossils were recorded from the deposit: the presumed bombine *Oligobombus cuspidatus* Antropov, 2014 and specimen NHMUK In.10012 (Megachilidae, incertae sedis) (Antropov et al. 2014).

The Florissant shale of Colorado (USA) (Swisher and Prothero 1990), situated near the Eocene-Oligocene boundary is approximately 34.0 Ma in age (Epis and Chapin 1974; Evanoff et al. 2001; Boyle et al. 2008; Mustoe 2008; Veatch and Meyer 2008). It produced a large number of the known bee fossils, most of which were described in the early part of the 20^th^ Century, with 36 specimens representing 34 species in 19 genera (Zeuner and Manning 1976; Michez et al. 2012). One possible bumble bee fossil was recorded: *Calyptapis florissantensis* Cockerell, 1906.

The deposits of BesKonak are from the Lower Miocene (Aquitanian, i.e., 22.5 Ma) and located in Anatolia, north of Ankara Province, Turkey (Paichelier et al. 1978). The only bombine fossils discovered in the deposits of BesKonak are *Oligoapis beskonakensis* Nel & Petrulevičius, 2003 and *Paraelectrobombus patriciae* Nel & Petrulevičius, 2003.

The Latah Formation encompasses the Lower to Middle Miocene (i.e., 21.3–12.1 Ma) of eastern Washington and northwestern Idaho (USA) (Berry 1929; Kirkham and Melville 1929; Gray and Kittleman 1967; Lewis 1969; Robinson 1991; Derkey et al. 2003). The only known bee fossils from this deposit are *Bombus proavus* Cockerell, 1931 and an undetermined megachiline specimen (Cockerell 1931; Engel 2004).

The deposits of the Most Formation at Bílina Mine date from the Lower Miocene (i.e., 20.0 Ma), in northern Bohemia (Czech Republic) (Kvaček 1998; Prokop and Nel 2000; Prokop et al. 2003; Kvaček et al. 2004; Knor et al. 2012). Two bee fossils have been reported from the deposits of the Most Formation: undetermined specimens of *Apis* and the bumble bee *B. trophonius* Prokop, Dehon, Michez & Engel, 2017 (Prokop et al. 2003; Prokop et al. 2017; Engel pers. obs.).

Krottensee, also in the Czech Republic, dates from the Lower Miocene (i.e., 18.0–17.0 Ma), and is also referred to as Mokřina (Bůžek et al. 1996; Mlíkovský 1996; Rojík 2004). A single bumble bee has been recovered from the deposits, *B. crassipes* Novák, 1878 (Novák 1878; Krzemiński and Prokop 2011).

The Randeck Maar deposits of the Lower-Middle Miocene (i.e.,18.0–16.0 Ma) are located in southwestern Germany, southeast of Stuttgart at the escarpment of the Swabian Alps (Heizmann 1983), and is the largest ancient Maar in that region (Köppen and Geiger 1928; Gregor 1986; Krautter and Schweigert 1991; Schweigert 1998; Lutz et al. 2000; Kottek et al. 2006). This fossil Lagerstätte contains exceptionally well-preserved flora and fauna (e.g., Armbruster 1938; Gregor 1986; Schawaller 1986; Ansorge and Kohring 1995; Kotthoff 2005; Kotthoff and Schmid 2005; Kotthoff et al. 2011). Several prominent bee fossils have been reported from Randeck Maar – *Apis armbrusteri* Zeuner, 1931 (Kotthoff et al. 2011), *B. randeckensis* Wappler & Engel, 2012 (Wappler et al. 2012), and *Halictus schemppi* (Armbuster, 1938) – and while those of *Bombus* and *Halictus* are each from single specimens, a plethora of honey bee workers have been recorded (Kotthoff et al. 2011).

The Middle Miocene sediments of the Shanwang Formation (17.0–15.2 Ma) are located in Linqu County, Shandong Province, China (Yang et al. 2007). Many insects have been listed from this deposit, including bees (Megachilidae, Apidae), and specifically the bumble bees *B. anacolus* Zhang, *B. luianus* Zhang, and *B. dilectus* Zhang (Zhang 1990; Zhang et al. 1994).

The Botchi Formation is from the Upper Miocene (i.e., 11.2–7.1 Ma) and is located on the left bank of the Botchi River in Russia (Khabarovsk Region) (Akhmetjev 1973). This formation has yielded various plants, fishes, Crustacea, and insects, including *B. vetustus* Rasnitsyn & Michener, 1991.

The deposit of Kumi (Euboea, Greece) is from the Middle-Upper Miocene (i.e., 11.2–7.1 Ma). Insects from the orders Coleoptera, Diptera, and Hymenoptera were discovered in the Kumi deposit, and these included *B. pristinus* Unger, 1867.

The Spanish deposit of La Cerdanya corresponds to Upper Miocene lacustrine beds (i.e., 10.0 Ma) located in Spain (Lleida, Bellver-en-Cerdaña) (Diéguez et al. 1996; Jiménez-Moreno et al. 2010). The flora and entomofauna are quite abundant and diverse (Peñalver-Molla et al. 1999; Arillo 2001) with a rather high occurrence of bees, although nearly all specimens belong to *Apis* (Nel et al. 1999). *Bombus cerdanyensis* Dehon, De Meulemeester & Engel, 2014 was described from this deposit (Dehon et al. 2014).

### Geometric morphometric analyses of forewing shape

We performed geometric morphometric analyses of the forewing shape in order to assess the taxonomic affinities of 12 bumble bee fossil species (13 specimens) showing well-preserved forewings (Fig. 1). This tool is useful in insect taxonomy for discriminating and diagnosing taxa at different levels (e.g., Pretorius 2005; Petit et al. 2006; Francoy et al. 2009, 2012; Sadeghi et al. 2009; Perrard et al. 2014; Van Cann et al. 2015), as well as in paleontology for assessing taxonomic affinities of fossils with contemporary and extinct taxa (e.g., Kennedy et al. 2009; Michez et al. 2009a; De Meulemeester et al. 2012; Wappler et al. 2012; Dehon et al. 2014, 2017; Dewulf et al. 2014; Perrard et al. 2016; Prokop et al. 2017). Several studies have demonstrated the utility of forewing shape analyses for diagnosing subgenera, species, and populations of bumble bees, depending on rearing conditions (e.g., Aytekin et al. 2007; Wappler et al. 2012; Barkan and Aytekin 2013; Gérard et al. 2018).

We used three different datasets to assess the taxonomic affinities of the fossils at different taxonomic levels. All three datasets represent a sampling of contemporary and extinct tribes with three submarginal cells, were largely assembled and analyzed in previous studies (i.e., Dehon et al. (2017) for the first dataset and Prokop et al. (2017) for the second and third datasets). We only (i) modified the classification of the species in the different datasets based on Bossert et al. (2019) and (ii) added the 13 fossils in each of these datasets. The first dataset included a comprehensive sampling of bee tribes in order to ensure correct tribal placement of the 13 fossils. This dataset consisted of 50 tribes representing 226 species and 979 specimens (refer to Dehon et al. (2017) for full details; Suppl material 1: Table S1). It also uncovered a group of six tribes (i.e., Ancylaini, Electrapini, Emphorini, Euglossini, Melikertini, and Tetrapediini) showing similar wing shapes to Bombini. We then used a second dataset with more extensive sampling within Bombini and these six similar tribes. This second dataset was assembled and tested by Prokop et al. (2017). It includes 841 specimens and represents all 15 subgenera and 210 species of extant bumble bees (80% of the total species diversity) as well as tribes Ancylaini, Electrapini, Emphorini, Euglossini, Melikertini, and Tetrapediini, representing a further 18 genera, 43 species, and 132 specimens altogether (Suppl material 2: Table S2). Finally, fossils confirmed to belong to Bombini using the first and second datasets were compared to a third dataset that only consists of the bumble bee specimens of the second dataset in order to assess the taxonomic affinities of the specimens with extant subgenera of *Bombus*. Dehon et al. (2017) (Suppl materials 3–5: Tables S3–S5) and Prokop et al. (2017) (Suppl materials 6–7: Tables S6–S7) demonstrated reliability for these datasets in classifying bee specimens based on forewing shape similarity relative to the reference datasets of forewings. Hence, the cross-validation allows us to be confident in the discrimination.

The potential effect of sexual dimorphism on subgeneric assignment using wing morphometry was tested by Wappler et al. (2012) for the subgenus Bombus s. str. For this subgenus, the results showed that sexual dimorphism had limited impact on subgeneric assignment. We tested it on four additional subgenera (based on 82 specimens from 12 species of four subgenera: *Bombias*, *Cullumanobombus*, *Melanobombus*, and *Mendacibombus*); the identification of the subgenera based on wing shape was again highly supported (Suppl material 7: Table S7). Therefore, to limit intraspecific variability in our dataset, we sampled female specimens only. We selected females because Bombini are mostly social species and workers (i.e., females) are the most abundant caste. Moreover, most of the known fossil specimens are females, although the holotype of *B. vetustus* is a male as evidenced by the lack of a corbicula, male flagellomeres, etc. (Rasnitsyn and Michener 1991).

Left forewings were photographed using an Olympus SZH10 microscope combined with a Nikon D200 camera. Photographs were then uploaded in the software tpsUTIL 1.69 (Rohlf 2013a). The forewing shape was captured by digitizing two-dimensional Cartesian coordinates of 18 landmarks on the wing veins and cells (Fig. 4) with the software tpsDIG version 2.27 (Rohlf 2013b). Position of the landmarks was based on Owen (2012) and other studies like De Meulemeester et al. (2012), Wappler et al. (2012), Dewulf et al. (2014), Dehon et al. (2014, 2017), Gérard et al. (2015), and Prokop et al. (2017). The two-dimensional configurations of the landmarks were superimposed using the GLS Procrustes superimposition in the software R version 3.0.2 (Rohlf and Slice 1990; Bookstein 1991; Adams and Otárola-Castillo 2013; R Development Core Team 2013). The closeness of the tangent space to the curved shape space was analyzed by calculating the least-squares regression slope and the correlation coefficient between the Procrustes distances (in the shape space) and the Euclidean distances (in the tangent space) (Rohlf 1999). This was calculated using the software tpsSMALL v1.25 (Rohlf 2013c).

### Shape discrimination at different taxonomic levels

Variation of shape in the dataset was explored with PCA analyses to visualize clustering and detect outliers (Fig. 5). Discrimination of the wing shape of the different taxa was assessed by Linear Discriminant Analyses (LDA) of the projected aligned configuration of landmarks like in Prokop et al. (2017). We performed three LDAs with the first dataset (Dehon et al. 2017, Suppl material 1: Table S1) with different levels a priori grouping (i.e., the groups are known a priori by the analysis): family, subfamily and tribe (LDA 1–3, Tables 1, Suppl materials 3–5). We did a fourth LDA analysis with the second dataset (i.e., bumble bees + six similar tribes, Suppl material 2: Table S2) with tribe level as a priori grouping (LDA 4, Table 1, Suppl material 6). Finally, we used a comprehensive sampling of extant bumble bees (i.e., third dataset, Suppl material 2: Table S2) for a fifth LDA considering the subgenus level as a priori grouping (LDA 5, Tables 1, Suppl material 7: Table S7). The LDA effectiveness was assessed by the percentages of individuals correctly classified to their original taxon (hit-ratio, HR) in a leave-one-out (LOO) cross-validation procedure based on the posterior probabilities of assignment (Suppl materials 3–7: Tables S3–S7). Given the observed scores of an “unknown”, the posterior probability (pp) equals the probability of the unit to belong to one group compared to all others. The unit is consequently assigned to the group for which the posterior probability is the highest (Huberty and Olejnik 2006). All discriminant analyses were performed using the R software (R Development Core Team 2013).

### Assignment of the bee fossils

Taxonomic affinities of the fossils were assessed based on the score in the predictive discriminant space of shapes. Aligned coordinates of the specimens from the three datasets (including the fossils) were used to calculate the same five LDA as presented in the previous section. Assignment of the fossils was estimated by calculating the Mahalanobis Distance between each fossil and group mean of each taxon and then assigning it to the nearest group in the discriminant shape of the LDA (Suppl materials 7–12: Tables S7–S12). Principal Component Analyses (PCA) were also computed to visualize shape affinities between the fossils and the extant groups in the second dataset (Fig. 5). Mahalanobis Distance is well suited for dealing with large datasets of close-relative taxa (Claude 2008).

## Results

### Geometric morphometric analyses

The assignment of each fossil was assessed in each dataset. When using the first dataset, all fossils were assigned to Apidae, more specifically to Apinae (except for the second specimen of *C. florissantensis* described by Cockerell (1908) and *B. vetustus*, both assigned to Eucerinae) and to Bombini (except for *B. dilectus* assigned to Tetrapediini, and *Oligobombus cuspidatus* and both specimens of *C. florissantensis*, all three assigned to Electrapini) (see LDA 1–3, Tables 1, Suppl materials 9–11). We then specifically assessed the assignment of each fossils based on the second and the third dataset. *Oligobombus cuspidatus* and both specimens of *C. florissantensis* were close to the shape space of contemporary Bombini, while being placed outside of contemporary Bombini and fossil Electrapini (Dataset 2, LDA 4). Among bombine subgenera, *Bombias* was most similar in forewing shape to *Oligobombus* and *Calyptapis* (Dataset 3, LDA 5). Based on our discriminant analyses, *Paraelectrobombus patriciae* was also similar to extant Bombini while being just outside of its shape space (Dataset 2, LDA 4). *Bombus beskonakensis* clustered within the shape space of extant Bombini (Dataset 2, LDA 4) and was similar to the subgenus Mendacibombus but outside the modern shape space of that subgenus (Dataset 3, LDA 5). The assignment of *B. trophonius* based on wing shape was already assessed in Prokop et al. (2017), this fossil clustered within contemporary *Cullumanobombus* (Dataset 3, LDA 5). The forewing shape of *B. randeckensis* was previously analyzed by Wappler et al. (2012) who proposed that the specimen was close to the subgenus Bombus s. str. Our analyses found a close similarity with *Cullumanobombus* (Dataset 3, LDA 5), while in Wappler et al. (2012) this subgenus was the fourth most similar subgenus to the fossil. This discrepancy may be explained by the fact that we used a larger and more diverse dataset, or possibly also because forewings were digitized by different experimenters in both studies. *Bombus luianus* clustered within Bombini based on its forewing shape (Dataset 2, LDA 4). Moreover, its forewing shape was similar to, but outside of modern *Melanobombus*, suggesting this fossil might be sister to extant *Melanobombus* (Dataset 3, LDA 5). *Bombus dilectus* did not cluster within the shape space of Bombini, but the tribe was the most similar (Dataset 2, LDA 4), but it is possible the published drawings are not entirely accurate. The most similar subgenus was the subgenus Bombias (Dataset 2, LDA 4), one of the most basal subgenera of the genus (Fig. 5). *Bombus anacolus* clustered just outside of crown-group Bombini while being quite similar to this tribe based on its forewing shape (Dataset 2, LDA 4), but again the published drawing is rather poor. Nonetheless, its forewing shape was similar to modern *Mendacibombus* (Dataset 3, LDA 5). *Bombus vetustus*, which is a male, was most similar to the tribe Bombini based on forewing shape but was placed outside of the shape space of modern Bombini (Dataset 2, LDA 4). Despite this, the most similar subgenus was *Bombias* (Dataset 3, LDA 5). There is only one specimen available of *B. pristinus*, which has incomplete wings. However, all landmarks are available except for number 16, whose position could be accurately estimated by the extension of cu-a and portion of vein A. We decided therefore to apply the same LDA analyses to the specimen, with the 18 landmarks. Our results found that this fossil clustered inside the shape space of Bombini (Dataset 2, LDA 4) and was similar to the subgenus Cullumanobombus (Dataset 3, LDA 5), although it would be worth reanalyzing this specimen with a dataset encompassing males from extant species in order to have greater confidence. Finally, the wing shape of *B. cerdanyensis* was first analyzed in Dehon et al. (2014). This specimen has incomplete wings; nonetheless, all landmarks are available except for numbers 17 and 18, but the position of the latter could be accurately estimated by the extension of cu-a and portion of vein A. Assignment of the fossil in the discriminant space did not allow a reliable subgeneric attribution. Herein, *B. cerdanyensis* is assigned to *Melanobombus* (Dataset 3, LDA 5). The subgeneric assignment of each fossil within *Bombus* s. l. through geometric morphometric analyses is summarized in Table 1.

### Systematics

In the following account of fossil bombine species, we have organized the taxa by general age, proceeding from oldest to youngest.

### Family Apidae Latreille

**Subfamily Apinae Latreille**

**Clade Corbiculata Engel**

**Stem-group Bombini Latreille**

#### Late Eocene

##### Genus Oligobombus

Taxon classificationAnimaliaHymenopteraApidae

Antropov, 2014

20364784-FA13-5678-85F0-C8C2E511A100

###### Type species.

*Oligobombus cuspidatus* Antropov, 2014, by original designation.

###### Diagnosis.

Sex unknown. Forewing distinctly pointed apically (apparently taphonomically altered); three submarginal cells of approximately equal sizes; marginal cell elongate, longer than distance between its apex and forewing tip, with apex roundly truncate; forewing distal membrane papillate; pterostigma short, with margin within marginal cell straight, approximately 4.0 times as long as prestigma; r-rs arising from distal part of pterostigma after its midpoint; 1rs-m straight; 2rs-m with posterior half curved apically; angle between 1rs-m and part of M inside third submarginal cell obtuse; first submarginal cell with an oblique translucent vein rs and not wider than second submarginal cell; second submarginal cell shorter than third marginal cell; third submarginal cell widest; 1m-cu slightly curved anteriorly, reaching second submarginal cell in its midpoint; 2m-cu curved anteriorly, reaching M basad 2rs-m; distance between anterior ends of 1m-cu and 2m-cu exceeding their length; basal vein slightly basad cu-a. See Antropov et al. (2014) for original diagnosis.

##### Oligobombus cuspidatus

Taxon classificationAnimaliaHymenopteraApidae

Antropov, 2014

10501FCC-FE5B-5ECF-9BCF-1A139D7A5D26

###### Holotype.

Sex unknown. NHMUK In.17349 (part and counterpart), Smith collection of the Natural History Museum (NHM, London, UK). Type specimen has been located and revised (Figs 1A, 3A).

###### Type strata and locality.

Late Eocene (i.e., 36.0 Ma), Insect Bed of the Bembridge Marls from the Isle of Wight, UK.

###### Diagnosis.

Owing to monotypy, the diagnosis for the species is identical to that of the genus (vide supra).

###### Description.

Part consists in middle and apical parts of right forewing; counterpart consists of middle part of right forewing; forewing distal membrane papillate; complete venation preserved; total forewing length 13.3 mm, maximum width 4.0 mm as preserved; basal vein length 2.3 mm, relatively straight and basad cu-a; cu-a length 0.3 mm; marginal cell length 4.0 mm, width 0.9 mm, apex roundly truncate; prestigma 0.2 mm; pterostigma length 0.8 mm; 1^st^ abscissa of Rs straight; 2^nd^ abscissa of Rs almost straight; 3Rs length approximately same as r-rs; 4Rs slightly longer than 3Rs; M+Rs length 1.2 mm; three submarginal cells; first submarginal cell length 1.5 mm (as measured from origin of Rs+M to juncture of r-rs and Rs), width 0.6 mm (as measured from Rs+M to pterostigma); second submarginal cell length 1.3 mm (as measured from juncture of Rs+M and M to juncture of Rs and 1rs-m), width 0.7 mm (as measured from midpoint on M between 1m-cu and 1rs-m to juncture of r-rs and Rs); third submarginal cell length 1.4 mm (as measured from juncture of 1rs-m and M to juncture of M and 2rs-m), width 1.0 mm (as measured from juncture of M and 2m-cu to juncture of 2rs-m and Rs); 1rs-m straight; 2rs-m posterior half curved apically; 1m-cu anterior half curved apically, reaching M approximately at midpoint between 2^nd^ abscissa of Rs and 1rs-m; 2m-cu basad 2rs-m. See Antropov et al. (2014) for original description.

###### Comments.

There is only one specimen, the holotype NHMUK In.17349, consisting of a part and counterpart. Antropov et al. (2014) described the specimen and considered it as possibly a member of Bombini. According to the original author, the forewing shape displays mixed features of Bombini, Electrapini, Electrobombini, Euglossini, and Melikertini (e.g., the forewing distal membrane being papillate is characteristic of Bombini, Electrobombini, and Euglossini, the shape of vein Rs displays mixed features reminiscent of the corbiculate tribes Bombini, Electrobombini, Electrapini (i.e., *Thaumastobombus* Engel, 2001), Euglossini, and Melikertini (i.e., *Melikertes* Engel, 1998 and *Succinapis* Engel, 2001), the submarginal cells are reminiscent of Electrapini, Electrobombini, and Euglossini, 1m-cu is reminiscent of Electrapini and Electrobombini, 2m-cu is reminiscent of Electrapini, Euglossini, and Melikertini). All in all, the specimen has a forewing venation with features that can be found in different extinct and extant tribes of Corbiculata, but that taken together do not occur in any of them. According to the Antropov et al. (2014), the fossil forewing venation is generally similar to extant species of Bombini, but the lack of features from the pro-, meso-, and metasoma prevents identification of its exact taxonomic affinities. Based on the general morphology and forewing shape affinities, *Oligobombus* is perhaps a stem-group bombine and we consider it as such for the moment. Further material and additional characters, ideally analyzed in a cladistic framework, are needed to corroborate this placement, or the species could have phylogenetic affinities with Electrobombini or Electrapini.

#### Eocene-Oligocene boundary

##### Genus Calyptapis

Taxon classificationAnimaliaHymenopteraApidae

Cockerell, 1906

EEF87154-D516-57A8-84DF-C00602C80D51

###### Type species.

*Calyptapis florissantensis* Cockerell, 1906, by original designation.

###### Diagnosis.

Three submarginal cells; third submarginal cell longest, shorter than combined length of first and second submarginal cells; first and second submarginal cells of more or less same size; first submarginal cell rounded; marginal cell wide, apex rounded and scarcely offset from anterior forewing margin; basal vein long and straight, slightly curved in its base, meeting M+Cu near juncture of cu-a with M+Cu; cu-a slightly curved; 1m-cu meeting M at middle of second submarginal cell; 2m-cu slightly curved and not in line with 2rs-m, positioned before crossing between 2rs-m and M; 2rs-m strongly arched; 2Rs scarcely arched basally; pterostigma relatively small. Pro- and mesosoma black; corbicula preserved; no alar papillae (or, more likely, not visible as preserved); forewing not colored. Similar in forewing venation to *Bombus* s. l. but differing from most species in the combination of a simultaneously distally bulging third submarginal cell (i.e., 2rs-m strongly arched), with a relatively unmodified second submarginal cell (i.e., 2Rs scarcely arched basally, a putatively plesiomorphic trait and somewhat similar to many euglossines), and broad marginal cell apex that is scarcely offset from anterior wing margin.

##### Calyptapis florissantensis

Taxon classificationAnimaliaHymenopteraApidae

Cockerell, 1906

825EFE15-4D17-5080-9680-375D9E0D0309

###### Holotype.

Sex unknown. MCZPALE 2008, collections of the Museum of Comparative Zoology (Harvard University, Cambridge, USA). Samuel Hubbard Scudder collection. Type specimen has been located and revised (Figs 1B, 3B).

###### Type strata and locality.

Eocene-Oligocene boundary (i.e., 34.0 Ma), the Florissant shale of Colorado, USA.

###### Diagnosis.

Owing to monotypy, the diagnosis for the species is identical to that of the genus (*vide supra*).

###### Description.

Integument of body black to dark brown as preserved (taphonomically altered); forewing venation brown to dark brown, membrane hyaline as preserved; forewing length 7.6 mm; maximum width approximately 2.5 mm as preserved; basal vein (1M) faintly arched at base, straight along length, basad 1cu-a by about twice vein width, faintly angled relative to 1Rs; Rs+M originating anteriad, 1Rs about as long as r-rs; pterostigma short, slightly longer than wide, border inside marginal cell slightly concave, prestigma very short, scarcely present, about as long as 2.5–3 times width of 1Rs; marginal cell length 2.2 mm, width 0.5 mm, tapering slightly across its length, free portion of cell subequal to portion bordering submarginal cells, apex rounded and offset from anterior wing margin by about vein width, not appendiculate; 2Rs weakly arched basally, comparatively straight; r-rs about as long as 3Rs; 4Rs slightly longer than 3Rs; three submarginal cells of comparatively similar sizes, albeit third slightly larger than first or second, but slightly shorter than combined lengths of first and second submarginal cells; first submarginal cell length 0.9 mm (as measured from origin of Rs+M to juncture of r-rs and Rs), width 0.4 mm (as measured from Rs+M to pterostigma); second submarginal cell length 0.7 mm (as measured from juncture of Rs+M and M to juncture of Rs and 1rs-m), width 0.4 mm (as measured from midpoint on M between 1m-cu and 1rs-m to juncture of r-rs and Rs); third submarginal cell length 0.9 mm (as measured from juncture of 1rs-m and M to juncture of M and 2rs-m), width 0.6 mm (as measured from juncture of M and 2m-cu to juncture of 2rs-m and Rs); 1rs-m weakly arched; 2rs-m strongly arched distally in posterior half, such that third submarginal cell is greatly bulged distally; 1m-cu distinctly angulate anteriorly near M, entering second submarginal cell slightly before cell’s midlength; 2m-cu weakly and gently arched apically, meeting third submarginal cell near cell’s apex, basad 2rs-m by about 2.5 times vein width; mesosoma length 4.4 mm as preserved; metasoma length 8.8 mm as preserved; total body length 15.2 mm as preserved. Specimen UCM 4415: left lateral view; pro-, meso-, and metasoma preserved, both forewings preserved; parts of right hindleg and foreleg preserved; forewing venation preserved; part of one antenna preserved. Specimen MCZPALE-2008: mesosoma preserved, as well as part of prosoma; right forewing visible. See Cockerell (1906, 1908c) for original description.

###### Comments.

*Calyptapis florissantensis* was first described based on a poorly preserved specimen collected by Samuel H. Scudder (MCZPALE 2008), and was first attributed to Eucerini by Cockerell (1906). The well-preserved second specimen (UCM 4415) was described by Cockerell (1908) and this permitted him to attribute both specimens to Bombini. However, he stated that the fossil differed from extant *Bombus* in the form of the second and third submarginal cells, thus suggesting it to be a member of a genus close to *Bombus* (Cockerell 1906, 1908; Zeuner and Manning 1976). Based on the general morphology and forewing shape affinities, *Calyptapis* is perhaps a stem-group bombine and we consider it as such for the moment, although a cladistic analysis encompassing additional characters is needed for a more definitive clarification of its phylogenetic affinities.

#### Oligocene-Miocene boundary

**Tribe Bombini Latreille**

**Genus *Bombus* Latreille**

##### Subgenus Paraelectrobombus

Taxon classificationAnimaliaHymenopteraApidae

Nel & Petrulevičius, 2003, nomen translatum

ACD7B324-5740-587A-A078-20C759C812C2

###### Type species.

*Paraelectrobombus patriciae* Nel & Petrulevičius, 2003.

###### Diagnosis.

Bombiform bee; pterostigma larger than prestigma; vein 1m-cu curved apically in its anterior half; vein r-rs reaching pterostigma at midpoint; second abscissa of Rs relatively straight; vein 2rs-m curved apically in its posterior half; vein 2m-cu slightly curved at midpoint, reaching M basad to 2rs-m; two tibial spurs; corbicula with setae longer than metatibia width. See Nel and Petrulevičius (2003) for original diagnosis.

##### Bombus (Paraelectrobombus) patriciae

Taxon classificationAnimaliaHymenopteraApidae

(Nel & Petrulevičius, 2003) comb. nov.

08DBFF23-5A6A-5F5F-9B6C-104A57567066

###### Holotype.

Female. MNHN-LP-R. 11187 (coll. Paichelier 1977), deposited in the Laboratoire de Palaeontologie, Muséum national d’Histoire naturelle, Paris, France. The type specimen was located, examined, and revised (Figs 1D, 3D).

###### Type strata and locality.

Oligocene-Miocene boundary, 22.5 Ma, volcano-sedimentary paleolake deposit, BesKonak Basin, Anatolia, Turkey.

###### Diagnosis.

Owing to monotypy, the diagnosis for the species is identical to that of the subgenus (vide supra).

###### Description.

Body poorly preserved and covered with long setae; forewing membrane hyaline and covered with small pilosity, venation similar to that of extant species of *Bombus* s. l.; forewing length 9.0 mm, maximum width approximately 3.4 mm as preserved; basal vein slightly curved at base, and slightly basad cu-a, length 1.9 mm; prestigma length 0.3 mm, width 0.2 mm; pterostigma length 0.6 mm, width 0.3 mm; marginal cell length 2.8 mm, width 0.6 mm, with apex narrowly rounded and detached from margin of forewing; 1^st^ abscissa of Rs straight; 2^nd^ abscissa of Rs curved basally in its last posterior part; r-rs almost straight; 3Rs smaller than r-rs; 4Rs approximately as long as r-rs; Rs+M straight and longer than r-rs; three submarginal cells of approximately equivalent size; first submarginal cell length 1.4 mm (as measured from origin of Rs+M to juncture of r-rs and Rs), width 0.6 mm (as measured from Rs+M to pterostigma); second submarginal cell length 1.1 mm (as measured from juncture of Rs+M and M to juncture of Rs and 1rs-m), width 0.6 mm (as measured from midpoint on M between 1m-cu and 1rs-m to juncture of r-rs and Rs); third submarginal cell length 1.0 mm (as measured from juncture of 1rs-m and M to juncture of M and 2rs-m), width 0.8 mm (as measured from juncture of M and 2m-cu to juncture of 2rs-m and Rs); 1rs-m almost straight; 2rs-m with anterior half curved apically; 1m-cu with anterior half curved apically, reaching M slightly before midlength between 2^nd^ abscissa of Rs and 1rs-m; 2m-cu slightly curved near midpoint, reaching M basad 2rs-m; prosoma length 3.0 mm as preserved; mesosoma length 4.5 mm as preserved; metatibia without basal plate, length 2.2 mm, width 0.6 mm; corbicula with long setae; metabasitarsus length 2.0 mm; width 1.0 mm, with auricle at base; metasoma not preserved. The taphonomy of the specimen does not allow us to ascertain the presence or absence of a transector. See Nel and Petrulevičius (2003) for original description.

###### Comments.

There is only one specimen, the holotype MNHN-LP-R. 11197. The fossil was initially described as *Paraelectrobombus patriciae* within the extinct tribe Electrobombini by Nel and Petrulevičius (2003), and was described as a bombine-like species with a wing venation similar to those of Bombini and Electrobombini. However, these authors stated that it was not possible to determine its exact relationship relative to Bombini and Electrobombini owing to the lack of information on its body structures such as the pretarsal claws and arolia. Based on the specimen’s forewing shape affinities, *Paralectrobombus* is assuredly an extinct taxon of Bombini, and likely within the genus *Bombus*. Based on our results, we hypothesize that this group may be sister to extant *Bombus* or a stem group to *Bombus*.

##### Subgenus Mendacibombus Skorikov, 1914

= *Oligoapis* Nel & Petrulevičius, 2003, syn. nov.

###### Bombus (Mendacibombus) beskonakensis

Taxon classificationAnimaliaHymenopteraApidae

(Nel & Petrulevičius, 2003) comb. nov.

FE696027-FDB2-5964-95F8-79E0A31272A6

####### Holotype.

Female worker. MNHN-LP-B.47780 (BK349, coll. Paichelier, in 1977), part and counterpart, deposited in the Laboratoire de Palaeontologie, Muséum national d’Histoire naturelle, Paris, France. Type specimen has been located and revised (Figs 1E, 3E).

####### Type strata and locality.

Oligocene-Miocene boundary, 22.5 Ma, volcano-sedimentary paleolake, BesKonak Basin, Anatolia, Turkey (Paichelier et al. 1978).

####### Diagnosis.

Habitus and hind and forewing venation similar to those of extant Bombini, with pterostigma short but longer than prestigma, and metatibial spurs not visible as preserved (seemingly obscured by leg orientation). Short process of proximal posterior corner of metabasitarsus apparently preserved. See Nel and Petrulevičius (2003) for original diagnosis.

####### Description.

Wing membrane red-brown, setose throughout; forewing length 15.0 mm; maximum width 5.2 mm as preserved; pterostigma slightly longer than prestigma, with posterior margin aligned with vein Sc+R; marginal cell with apex closed by strong vein; three submarginal cells of approximately same size; basal vein long, oblique and slightly curved in its base, slightly basad cu-a; cu-a straight; 1m-cu strongly curved apically in its anterior half, reaching second submarginal cell near midpoint; 2m-cu curved apically, reaching M basad to 2rs-m; second abscissa of Rs slightly double-curved; 1rs-m almost straight; 2rs-m with posterior half curved apically; prosoma length 6.3 mm, covered with long and dark hair; mouthparts not preserved, except for galea which is elongate; antennae approximately 3.5 mm long, with nine or ten visible flagellomeres, scape and pedicel poorly preserved; mesosoma length 8.0 mm, height 5.0 mm; metafemur length 4.2 mm, width 1.4 mm, with long curved hair; metatibia length 4.5 mm, width 1.8 mm, with corbicula; metabasitibial plate absent; metatibial spurs not visible as preserved (apparently owing to leg orientation); metabasitarsus length 2.7 mm, width 1.7 mm, with auricle preserved; arolia and claws not visible as preserved; metasoma length 9.0 mm, height 4.5 mm, covered with short setae. See Nel and Petrulevičius (2003) for original description.

####### Comments.

The fossil was first described as *Oligoapis beskonakensis* by Nel and Petrulevičius (2003). The specimen is remarkably similar to extant Bombini in terms of its habitus and wing venation. However, the authors decided to place it in a separate genus of an undetermined corbiculate tribe owing to its pterostigma smaller than the prestigma, and by the putative absence of metatibial spurs. The absence of metatibial spurs is merely due to the lack of preservation and not to the definitive absence of spurs, and therefore this character cannot be evaluated. The metatibia is preserved with its outer surface exposed and the presence of spurs (particularly if they were reduced in size) on the inner anterior angle could not be observed in this orientation. In extant species of *Mendacibombus*, females are characterized by a few long bristles emerging from the outer surface of the metatibia, by a metatibia with the outer surface imbricate, i.e. coarsely sculptured, as well as by an unusually short (i.e., for *Bombus* s. l.) process of the proximal posterior corner of the metabasitarsus (Williams et al. 2008, 2016). In the fossil, the long bristles emerging from the outer surface of the metatibia are not visible, while the short process of the proximal posterior corner of the metabasitarsus appears to be present. Furthermore, it is challenging to assess if the metatibia outer surface is coarsely sculptured due to the taphonomy of the specimen.

We consider the fossil as a stem group within *Mendacibombus* and thus synonymize Oligoapis under that subgenus. Like Oligoapis, *Mendacibombus* has a relatively reduced pterostigma, further emphasizing the similarity between these groups. Interestingly, this species from the Oligocene-Miocene boundary (i.e., 22.5 Ma) comes from a deposit near the estimated Old World origin of this subgenus (Williams et al. 2016). Because of the overall morphological assessment we place the species as a stem group within *Mendacibombus*.

#### Lower Miocene

##### Subgenus Cullumanobombus Vogt, 1911

###### Bombus (Cullumanobombus) trophonius

Taxon classificationAnimaliaHymenopteraApidae

Prokop, Dehon, Michez & Engel, 2017

7C3C02D5-6CD4-557C-B95B-C15FC56225DA

####### Holotype.

**Figure 2.**
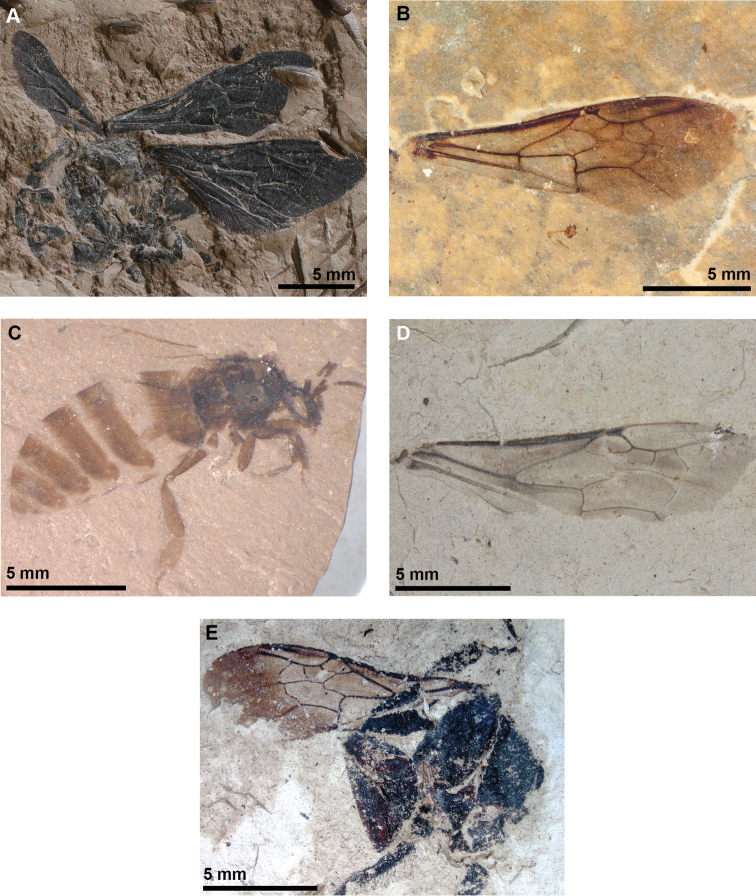
Representative fossil bumble bees **A**Bombus (Cullumanobombus) trophonius (photograph by Jakup Prokop) **B**B. (Cullumanobombus) randeckensis (photograph by Torsten Wappler) **C***B. vetustus* (photograph by Alexandr P. Rasnitsyn) **D**B. (Cullumanobombus) pristinus (photograph by Irene Zorn and Monika Brüggeman-Ledolter) **E**B. (Melanobombus) cerdanyensis (photograph by Thibaut De Meulemeester).

**Figure 3.**
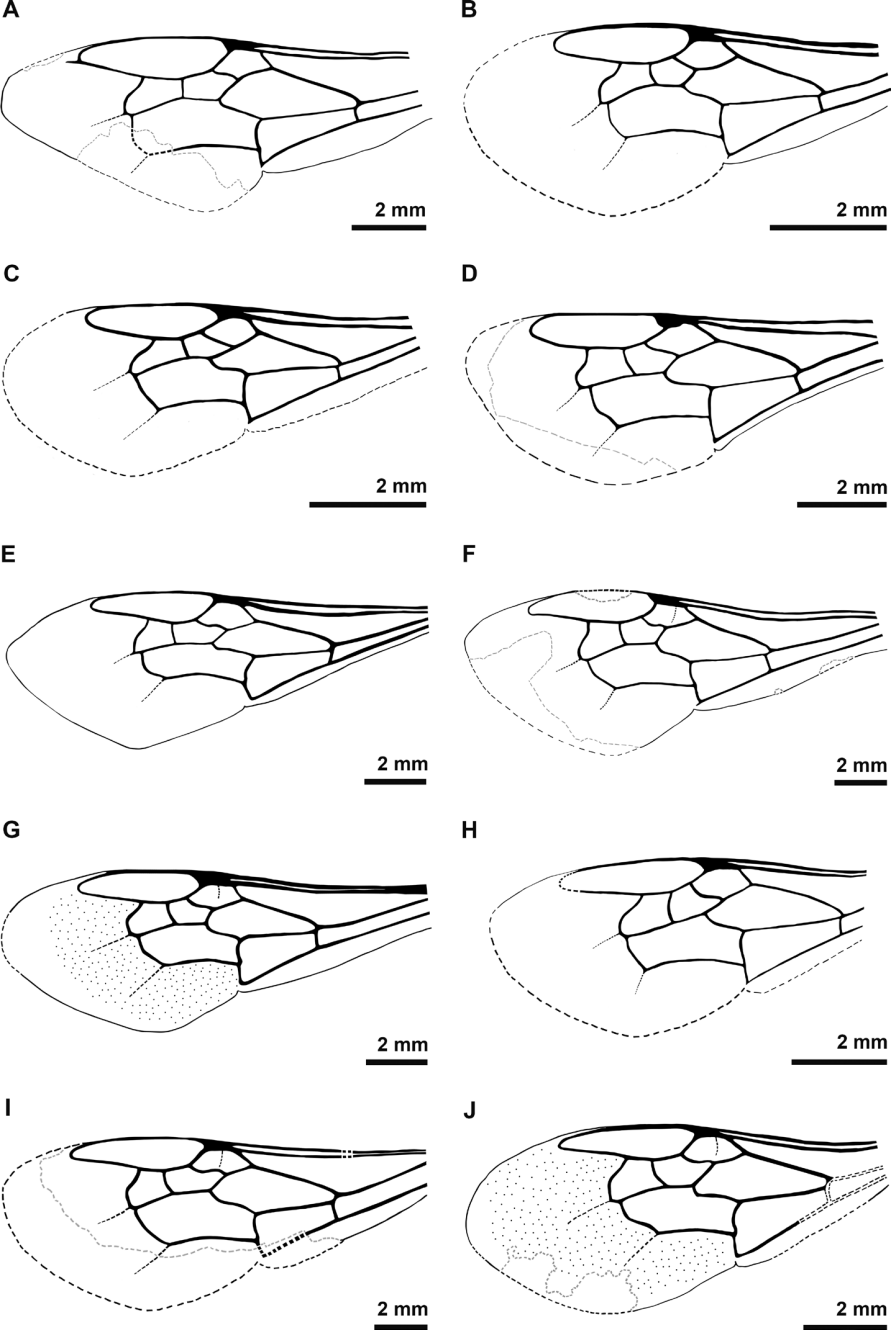
Forewing drawings of the fossil bumble bees studied herein. Some forewings were mirrored to enable comparison across all specimens **A***Oligobombus cuspidatus* (mirrored) **B** Holotype of *Calyptapis florissantensis* (mirrored) **C***C. florissantensis***D**Bombus (Paraelectrobombus) patriciae (mirrored) **E**B. (Mendacibombus) beskonakensis**F**B. (Cullumanobombus) trophonius (mirrored) **G**B. (Cullumanobombus) randeckensis (mirrored) **H***B. vetustus***I**B. (Cullumanobombus) pristinus (mirrored) **J**B. (Melanobombus) cerdanyensis.

Female. ZD0003 (coll. Bílina mine). Type specimen has been located and revised (Figs 2A, 3F).

####### Type strata and locality.

Lower Miocene (i.e., 20.0 Ma), Clayey Superseam Horizon, Bílina mine, Czech Republic.

####### Diagnosis.

The fossil has a wing pattern most similar to B. (Cullumanobombus) rufocinctus Cresson (Milliron 1973; Williams et al. 2014). Moreover, both species display a similar combination of 3Rs about as long as r-rs but shorter than 4Rs, a basal vein basad 1cu-a, a vein 2Rs arched posteriorly but not as greatly prolonged proximally as in several other species of *Cullumanobombus* (e.g., Milliron 1971), and a vein 1m-cu entering second submarginal cell near midpoint. However, the convex pterostigmal border within the marginal cell, less apically narrowed marginal cell, and less arched 2rs-m minimally serve to distinguish the fossil species from *B. rufocinctus*. See Prokop et al. (2003) and Prokop et al. (2017) for original diagnosis.

####### Description.

Wings and integument black as preserved; forewing total length 14.6 mm; maximum width 5.10 mm; basal vein weakly arched basally, comparatively straight along length, basad cu-a by about vein width, in line with 1Rs; M+Rs originating anteriad, 1Rs slightly shorter than r-rs; pterostigma short, slightly longer than wide, tapering inside of marginal cell, border inside marginal cell convex, prestigma nearly as long as pterostigma; marginal cell length 5.1 mm, width 1.1 mm, free portion slightly shorter than portion bordering submarginal cells, apex rounded and offset from anterior wing margin by much more than vein width, not appendiculate; 2Rs strongly arched basally and slightly arched outward; r-rs about as long as 3Rs; 4Rs slightly longer than 3Rs; three submarginal cells of approximately same sizes, albeit third slightly larger than first or second; first submarginal cell length 0.9 mm (as measured from origin of M+Rs to juncture of r-rs and Rs), width 1.0 mm (as measured from Rs+M to pterostigma); second submarginal cell length 1.3 mm (as measured from juncture of Rs+M and M to juncture of Rs and 1rs-m), width 0.9 mm (as measured from midpoint on M between 1m-cu and 1rs-m to juncture of r-rs and Rs); third submarginal cell length 1.6 mm (as measured from juncture of 1rs-m and M to juncture of M and 2rs-m), width 1.2 mm (as measured from juncture of M and 2m-cu to juncture of 2rs-m and Rs); 1rs-m straight; 2rs-m arched distally in posterior half; 1m-cu distinctly angulate anteriorly near M, entering second submarginal cell near cell’s midlength; 2m-cu slightly arched apically, meeting third submarginal cell at cell’s apical fifth of length. Hind wing length 9.4 mm, width 2.6 mm. Preserved portion of mesosoma and legs difficult to describe, although portion of metatibial corbicula preserved (basal quarter to third), and sclerites with numerous, long setae. See Prokop et al. (2003) and Prokop et al. (2017) for original description.

####### Comments.

The specimen was first reported as *Bombus* sp. in Prokop et al. (2003). Prokop et al. (2017) demonstrated that the fossil clustered within contemporary *Cullumanobombus* and formally described the species. Although the majority of contemporary species of *Cullumanobombus* are found in the New World and a few species in the Old World, Hines (2008) estimated that the subgenus originated around 20.0–15.0 Ma in the Palearctic. Our result, as well as that of Prokop et al. (2017), is consistent with Hines (2008) as the fossil specimen was found in the Lower Miocene (i.e., 20.0 Ma) deposits of Bílina Mine in northern Bohemia (Czech Republic).

###### Bombus (Cullumanobombus) randeckensis

Taxon classificationAnimaliaHymenopteraApidae

Wappler and Engel in Wappler et al. (2012)

8B21711F-88C2-52C8-8A97-5A96995A551D

####### Holotype.

Sex unknown. The fossil consists of an isolated forewing. SMNS 68000/28 (old Armbruster collection No. A5119). Conserved in the Staatliches Museum für Naturkunde, Stuttgart, Germany. Type specimen has been located and revised (Figs 2B, 3G).

####### Type strata and locality.

Randeck Maar, southeast of Stuttgart, Swabian Alb; Early Miocene, i.e., 16.0–18.0 Ma (Burdigalian, Karpatian, MN 5).

####### Diagnosis.

Bombiform bee; infuscate area in marginal cell extends entire length of anterior half of marginal cell; forewing venation strictly similar to that of an extant bumble bee, with transector visible on both forewings. See Wappler et al. (2012) for original diagnosis.

####### Description.

Forewing length 14.3 mm, maximum width 5.0 mm; marginal cell length 3.9 mm; basal vein almost straight, slightly curved in its base, slightly basad cu-a; vein cu-a straight; three submarginal cells; first submarginal cell length 1.7 mm (as measured from origin of M+Rs to juncture of r-rs and Rs), width 0.8 mm (as measured from M+Rs to pterostigma); second submarginal cell width 0.7 mm (as measured from midpoint on M between 1m-cu and 1rs-m to juncture of r-rs and Rs); third submarginal cell length 1.3 mm (as measured from juncture of 1rs-m and M to juncture of M and 2rs-m), width 1.1 mm (as measured from juncture of M and 2m-cu to juncture of 2rs-m and Rs); height of second medial cell 1.1 mm (as measured from Cu1 to juncture of 1m-cu and M); 1^st^ abscissa of Rs almost straight; 2^nd^ abscissa of Rs with anterior half curved apically; r-rs almost straight; M+Rs straight and longer than r-rs; 3Rs almost as long as r-rs; 4Rs slight smaller than M+Rs; 1rs-m almost straight; 2rs-m with posterior half curved apically; 1m-cu curved apically in last anterior part, reaching second submarginal cell before midpoint; 2m-cu slightly curved, reaching M basad to 2rs-m. See Wappler et al. (2012) for original description.

####### Comments.

The fossil was discovered in the Lower Miocene (i.e., 18.0–16.0 Ma) deposits of Randeck Maar, Germany, an age and locality in general accord with the estimate that *Cullumanobombus* originated between 20.0–15.0 Ma in the Old World. Based on the forewing shape affinities and the general morphological assessment, *B. randeckensis* is likely an extinct species of *Cullumanobombus*, like *B. trophonius*.

#### Middle-Lower Miocene

##### “Bombus” luianus

Taxon classificationAnimaliaHymenopteraApidae

Zhang, 1990, species inquirenda

2D574CEF-60CB-585D-817B-D1EAC4DEDA90

###### Holotype.

Female. Specimen n°82771. Plate XXXIII-1, fig. 164 from Zhang et al. (1994), plate I-1, 2 from Zhang (1990). The type material from Shanwang was not available for study and we have, therefore, had to base our information on this and the following two species (vide infra) on the original Chinese descriptions, the rather poor original photographs, and the tenuously accurate line drawings in these publications. Accordingly, our evaluation of *B. luianus*, *B. dilectus*, and *B. anacolus* has been considerably hampered.

###### Type strata and locality.

Middle Miocene (i.e., 17.0–15.2 Ma), deposit of the Shanwang Formation, large lacustrine and lithified deposit, with diatomaceous and tuffaceous mudstone. Located in Linqu County, Shanwang Province, China.

###### Description.

Taken from Zhang (1990) and Zhang et al. (1994): Prosoma poorly preserved; meso- and metasoma preserved; mesosoma stout, setose, and dark; metasoma dark, reddish-brown near apex, displaying five segments, suboval in shape, little longer than wide, distinctly narrower than mesosoma; forewing membrane brown and transparent, venation dark brown; metatibia widening posteriorly, displaying two strong spurs, outer margin covered with strong coarse setae; metabasitarsus flat, rectangular, truncated at both ends, nearly as wide as distal part of metatibia; tarsomere IV displaying pair of spur-like bristles distally; inner margin of pretarsal claw displaying single tooth at midlength; forewing length approximately 14.0 mm, maximum width approximately 4.5 mm as preserved; basal vein relatively straight and almost in line with cu-a; cu-a almost straight; 1^st^ abscissa of Rs straight; 2^nd^ abscissa of Rs curved anteriorly; r-rs curved; Rs+M straight and shorter than r-rs; 3Rs almost straight and as long as r-rs; 4Rs almost straight and longer than r-rs; marginal cell length approximately 4.0 mm, width 0.8 mm; three submarginal cells; 1rs-m slightly curved apically near midpoint; 2rs-m curved apically in its posterior half; 1m-cu almost straight and reaching M near midpoint between 2^nd^ abscissa of Rs and 1rs-m; 2m-cu slightly curved and reaching M basad to 2rs-m; hind wing length 8.6 mm; total body length approximately 13.0 mm, width approximately 8.0 mm as preserved. The original description and figure do not display a transector vein. See Zhang (1990) and Zhang et al. (1994) for original descriptions.

###### Comments.

According to Zhang (1990), the fossil species is closely similar to B. (Bombus) tunicatus Smith, 1852 (extant species distributed in Himalaya), but differs from it in that the mesosoma is narrower than the mesosoma, and not so massive as is usual for the genus; the spurs becoming shorter; vein 1m-cu meeting second submarginal cell at midlength; and veins M+Cu and M of hind wing aligned in a straight line. The validity of these features for distinguishing the species remains unclear. Our morphometric study showed a similar shape with the subgenus Melanobombus. It is estimated that *Melanobombus* originated between the Lower and Middle Miocene, while the fossil was discovered in the Middle Lower Miocene (i.e., 17.0–15.2 Ma) deposits of Shandong, China (Zhang 1990; Zhang et al. 1994). The results based on geometric morphometric analyses for this species could be wrong, since they were based on Zhang’s drawings and not on a picture or on examination of the holotype. Given this, we consider the fossil as species inquirenda.

##### “Bombus” dilectus

Taxon classificationAnimaliaHymenopteraApidae

 Zhang, 1994, species inquirenda

F88F1DF6-214B-5025-A1B8-CE6BA0AF1F1A

###### Holotype.

Female. Plate XXXIII-3, figs 168, 169 from Zhang et al. (1994). We were not able study the holotype (see comment under *B. luianus*, vide supra).

###### Type strata and locality.

Middle Miocene (i.e., 17.0–15.2 Ma), deposit of the Shanwang Formation, large lacustrine and lithified deposit, with diatomaceous and tuffaceous mudstone. Located in Linqu County, Shanwang Province, China.

###### Description.

Taken from Zhang et al. (1994): Forewing and hind wing membrane papillate distally; forewing membrane dark brown; forewing length more than 15.0 mm, maximum width more than 6.0 mm as preserved; basal vein slightly curved, basad cu-a; cu-a very slightly curved apically; marginal cell length approximately 5.0 mm; 1^st^ abscissa of Rs slightly curved apically near midpoint; 2^nd^ abscissa of Rs curved apically near midpoint; r-rs straight; Rs+M straight and longer than r-rs; 3Rs straight and smaller than r-rs; 4Rs almost as long as Rs+M; three submarginal cells; 1rs-m straight; 2rs-m curved apically in its posterior half; 1m-cu straight, reaching M near midpoint between 2^nd^ abscissa of Rs and 1rs-m; 2rs-m curved and reaching M basad to 2rs-m; total body length approximately less than 20.0 mm as preserved. The original description and figure do not display a transector vein. See Zhang et al. (1994) for original description.

###### Comments.

The specimen was first described as *B. dilectus* by Zhang et al. (1994) and was stated to be similar to *B. anacolus* in that the wing color of both fossil species is rather dark and not transparent, or at most semi-transparent at the wing margins, a character differing from that of living species. However, some extant species display fairly dark wings (e.g., B. (Melanobombus) simillimus Smith, 1852). The authors also stated that the wings and body color of *B. dilectus* are darker than *B. anacolus.* As observed for *B. luianus*, results based on geometric morphometric analyses for this species (i.e., similarity to subgenus Bombus) could be wrong, since it was based on Zhang’s drawings. Given this, we consider this fossil as species inquirenda.

##### “Bombus” anacolus

Taxon classificationAnimaliaHymenopteraApidae

 Zhang, 1994, species inquirenda

6597B833-CB6A-5AB5-815B-ACDC70EFFA4B

###### Holotype.

Female. Plate XXXIII-2, figs 165, 166, 167 in Zhang et al. (1994). We were not able study the holotype (see comment under *B. luianus*, vide supra).

###### Type strata and locality.

Middle Miocence (i.e., 17.0–15.2 Ma), deposit of the Shanwang Formation, large lacustrine and lithified deposit, with diatomaceous and tuffaceous mudstone. Located in Linqu County, Shanwang Province, China.

###### Description.

Taken from Zhang et al. (1994): Forewing blackish brown, opaque; forewing and hind wing papillate distally; forewing length approximately 15.0 mm, maximum width approximately 6.00 mm as preserved; basal vein relatively straight and basad cu-a; cu-a almost straight; marginal cell length almost 5.0 mm, width 1.1 mm; 1^st^ abscissa of Rs almost straight; 2^nd^ abscissa of Rs slightly curved near midpoint; r-rs straight; Rs+M straight and longer than r-rs; 3Rs straight and smaller than r-rs; 4Rs straight and approximately as long as Rs+M; three submarginal cells, second smallest; 1rs-m straight; 2rs-m curved apically in its posterior half; 1m-cu relatively straight, reaching M near midpoint between 2^nd^ abscissa of Rs and 1rs-m; 2m-cu curved apically, reaching M basad to 2rs-m; total body length approximately 13.0 mm as preserved (large part of metasoma missing). See Zhang et al. (1994) for original description.

###### Comments.

The specimen was described as *B. anacolus* by Zhang et al. (1994), and considered to be close to *B. luianus*, a species collected from the same deposit. Based on geometric morphometric analyses this species is similar to *Mendacibombus*, and it could be a relative of this subgenus. This hypothesis is supported by the fact that *Mendacibombus* is estimated to have originated around the Eocene-Oligocene boundary (i.e., 34 Ma) in the Old World (Hines 2008), while the fossil was discovered in the Middle Lower Miocene (i.e., 17.0–15.2 Ma) deposit of Shandong in China (Zhang 1990; Zhang et al. 1994). Moreover, the crown age of extant members of *Mendacibombus* apparently diversified during the Late Miocene (i.e., 8 Ma). As observed for *B. luianus* and *B. dilectus*, results based on geometric morphometric analyses for this species could be wrong since it was based on Zhang’s drawings. Given this, we consider the fossil as species inquirenda.

#### Upper Miocene

##### “Bombus” vetustus

Taxon classificationAnimaliaHymenopteraApidae

 Rasnitsyn & Michener, 1991, species inquirenda

CCBB3809-1657-5AD6-9EAC-DF0C7B692E4B

###### Holotype.

Male. #2054/229, part and counterpart impressions of an entire male, deposited in the Palaeontological Institute, Russian Academy of Science, Moscow. Type specimen was located and revised (Figs 2C, 3H).

###### Type strata and locality.

Upper Miocene (i.e., 11.2–7.1 Ma), Botchi Formation, located on the left bank of the Botchi River, Russia.

###### Description.

Male: Forewing length 10.4 mm as preserved; basal vein long and slightly basad cu-a; cu-a straight; marginal cell length approximately 3.3 mm, width approximately 0.7 mm as preserved; 1^st^ abscissa of Rs straight; 2^nd^ abscissa of Rs relatively straight; r-rs almost straight; Rs+M slightly curved and slightly longer than r-rs; 3Rs smaller than r-rs; 4Rs slightly longer than Rs+M; three submarginal cells; first submarginal cell length 1.3 mm (as measured from origin of Rs+M to juncture of r-rs and Rs), width 0.6 mm (as measured from Rs+M to pterostigma); second submarginal cell length 1.1 mm (as measured from juncture of Rs+M and M to juncture of Rs and 1rs-m), width 0.6 mm (as measured from midpoint on M between 1m-cu and 1rs-m to juncture of r-rs and Rs); third submarginal cell length 1.2 mm (as measured from juncture of 1rs-m and M to juncture of M and 2rs-m), width 0.9 mm (as measured from juncture of M and 2m-cu to juncture of 2rs-m and Rs); 2rs-m with posterior half curved apically, 1m-cu reaching M near midpoint; 2m-cu curved and reaching M basad to 2rs-m; prosoma length 3.9 mm; profemur length 1.9 mm; protibial length 1.8 mm; basitarsus length 1.6 mm; setae of pro- and mesosoma dark; total body length 19.2 mm as preserved. See Rasnitsyn and Michener (1991) for original description.

###### Comments.

Given that this is a male specimen, further work is needed with comparisons of its forewing shape with a diverse dataset based on males. In addition, the venation is incompletely preserved and so hopefully further and more complete material will be discovered.

##### Subgenus Cullumanobombus Vogt, 1911

###### Bombus (Cullumanobombus) pristinus

Taxon classificationAnimaliaHymenopteraApidae

Unger, 1867

D5E9E013-76D1-5715-B87F-571016878232

####### Holotype.

Inventory number GBA 1867/004/0004. Sex unknown. The holotype is currently deposited in the Geologische Bundesanstalt (Vienna, Austria). Type specimen has been located and revised (Figs 2D, 3I).

####### Type strata and locality.

Upper Miocene (i.e., 11.2–7.1 Ma), Kumi deposit, Euboea Island (Euboea, Greece).

####### Diagnosis.

Basal vein long and almost straight, basad to apically curved cu-a; pterostigma slightly longer than prestigma: second abscissa of Rs with anterior half curved apically; three submarginal cells of approximately same size; 1rs-m almost straight; 2rs-m posterior half curved apically; 1m-cu with anterior half curved apically, reaching second submarginal cell slightly before midpoint; 2m-cu very slightly curved, reaching M basad to 2rs-m.

####### Description.

Forewing length approximately 16.0 mm, maximum width 4.3 mm as preserved; forewing membrane hyaline, venation black becoming grey when reaching apex of forewing; marginal cell length 4.9 mm, width 1.2 mm; basal vein long and almost straight, basad cu-a; vein cu-a curved apically; pterostigma slightly longer than prestigma; three submarginal cells; first submarginal cell length 2.1 mm (as measured from origin of Rs+M to juncture of r-rs and Rs), width 0.9 mm (as measured from Rs+M to pterostigma); second submarginal cell length 2.2 mm (as measured from juncture of Rs+M and M to juncture of Rs and 1rs-m), width 0.9 mm (as measured from midpoint on M between 1m-cu and 1rs-m to juncture of r-rs and Rs); third submarginal cell length 1.6 mm (as measured from juncture of 1rs-m and M to juncture of M and 2rs-m), width 1.3 mm (as measured from juncture of M and 2m-cu to juncture of 2rs-m and Rs); second abscissa of Rs with anterior half curved apically; 1rs-m almost straight; 2rs-m posterior half curved apically; 1m-cu with anterior half curved apically, reaching second submarginal cell slightly before midpoint; 2m-cu very slightly curved, reaching M basad to 2rs-m. It seems that a transector vein is visible on the first submarginal cell, but it might be an artefact created by the taphonomic alteration of the specimen. See Unger (1867) for original description.

####### Comments.

The type of *B. pristinus* consists of just one right forewing. The specimen was described and illustrated by Unger (1867). The illustration, displaying a left forewing, is reversed left to right. Unger attributed the species to Regenhofer but, according to Rasnitsyn and Michener (1991), the latter appears not to have written the comments or prepared the illustration of Unger’s work, thus making them conclude that the name must be attributed to Unger. Based on morphological and geometric morphometric analyses, it is likely that this fossil is an extinct species of *Cullumanobombus*.

##### Subgenus Melanobombus Dalla Torre, 1880

###### Bombus (Melanobombus) cerdanyensis

Taxon classificationAnimaliaHymenopteraApidae

Dehon, De Meulemeester & Engel, 2014

81A479F7-619E-50A8-8342-300A233BDFB5

####### Holotype.

Sex unknown. Conserved in the Paleontology department collection, Muséum national d’Histoire naturelle, Paris, France. The fossil consists of a part and counterpart. Type specimen has been located and revised (Figs 2E, 3I).

####### Type strata and locality.

Late Miocene (i.e., 10.0 Ma), lacustrine beds of Cerdanya, Spain.

####### Diagnosis.

Forewing membrane with alar papillae beyond apical crossveins; membrane infuscate, particularly in area beyond apical crossveins and along anterior borders of radial and marginal cells; pterostigma small, trapezoidal, not larger relative to prestigma and width not much shorter than length; marginal cell longer than distance from apex to forewing tip, tapering in width across its length, with apex acutely rounded and slightly offset from forewing margin; three submarginal cells of approximately same size, anterior borders of second and third submarginal cells subequal; 1m-cu angulate anteriorly, meeting second submarginal cell near midpoint; 2m-cu slightly arched, meeting third submarginal cell in apical fifth; mesotibia five times longer than wide; transector vein visible in the first submarginal cell. See Dehon et al. (2014) for original diagnosis.

####### Description.

Fossil compressed in apparently dorsal oblique view, with left forewing outstretched; right forewing not preserved; hind wings not preserved; prosoma not preserved; mesosoma and metasoma incomplete and damaged; mid and hind legs preserved, partially overlapping forewing; right profemur length 1.4 m, width 0.8 mm as preserved; left mesofemur length 3.6 mm, width 0.9 mm; mesotibia length 3.0 mm, width 0.6 mm; mesobasitarsus length 3.2 mm, width 0.9 mm; remaining tarsomeres and pretarsal claws well preserved; pretarsal claws apparently not toothed as preserved; right mesofemur length 3.5 mm, width 0.5 mm; mesotibia length 2.0 mm, width 0.4 mm as preserved; left forewing length 13.3 mm, maximum width 4.6 mm; three submarginal cells of similar size; first submarginal cell length 1.5 mm (as measured from origin of Rs+M to juncture of r-rs and Rs), heigth 0.7 mm (as measured from Rs+M to pterostigma); second submarginal cell length 1.5 mm (as measured from juncture of Rs+M and M to juncture of Rs and 1rs-m), height 0.8 mm (as measured from midpoint on M between 1m-cu and 1rs-m to juncture of r-rs and Rs); third submarginal cell length 1.3 mm (as measured from juncture of 1rs-m and M to juncture of M and 2rs-m), height 1.1 mm (as measured from juncture of M and 2m-cu to juncture of 2rs-m and Rs); first medial cell length 3.4 mm (as measured from juncture of M+Cu and Cu to juncture of 1m-cu and M), height 1.2 mm (as measured from juncture of M and Rs+M to midpoint on Cu between M+Cu and 1m-cu); pterostigma length 0.9 mm; marginal cell length 3.4 mm with apex rounded, offset from anterior wing margin, not appendiculate; 1m-cu strongly curved, meeting second submarginal cell near midpoint; 2m-cu slightly arched, meeting third submarginal cell in apical fifth; metasoma width 5.8 mm as preserved; first two segments visible, first segment length 1.8 mm, second segment length 1.2 mm as preserved. See Dehon et al. (2014) for original description.

####### Comments.

The attribution based on geometric morphometric analysis (i.e., *Melanobombus*) is consistent with the timing and geographic origin of the subgenus proposed by Hines (2008). Indeed, the fossil was found in the Upper Miocene (i.e., 10.0 Ma) deposit of La Cerdanya in Spain, while *Melanobombus* is estimated to have originated between 20.0–15.0 Ma in the Old World. The relative sizes of the prestigma and pterostigma exclude a placement in the Electrobombini (although the presence or absence of a jugal lobe in the hind wing cannot be determined in the holotype). The forewing is apically papillate (as in Bombini), and the marginal cell is not appendiculate and 1m-cu is strongly angulate together suggesting the species does not belong to the Electrapini or Melikertini (although some melikertines have 1m-cu more angulate, such as *Melissites trigona* Engel, 1m-cu is always much shorter and not as long as in Bombini or Euglossini; a long 1m-cu is more plesiomorphic among Corbiculata). Indeed, the forewings of the present fossil are distinctly *Bombus*-like: presence of papillae, general infuscation of the membrane, three submarginal cells of relatively similar size (albeit the latter character is assuredly plesiomorphic). Based on the specimen morphology and forewing shape affinities, the fossil is likely an extinct species of *Melanobombus*.

## Discussion

### Geometric morphometrics of forewing shape to discriminate taxa

As shown in Michez et al. (2009b), De Meulemeester et al. (2012), Wappler et al. (2012), Dehon et al. (2014, 2017), Dewulf et al. (2014), and Prokop et al. (2017), geometric morphometric analyses of forewing shape provide a robust tool for assessing the taxonomic affinities of bee fossils with contemporary taxa and insights into bee evolution. We additionally demonstrate herein that the Hit Ratios for subgeneric level assessments were high for the genus *Bombus*. However, we need to combine geometric morphometrics of forewing shape with morphological features (e.g., pilosity, leg morphology, head and mouthpart characters, etc.) to get more powerful results, but such morphological characters are either limited or lacking with the current impression fossils.

**Figure 4.**
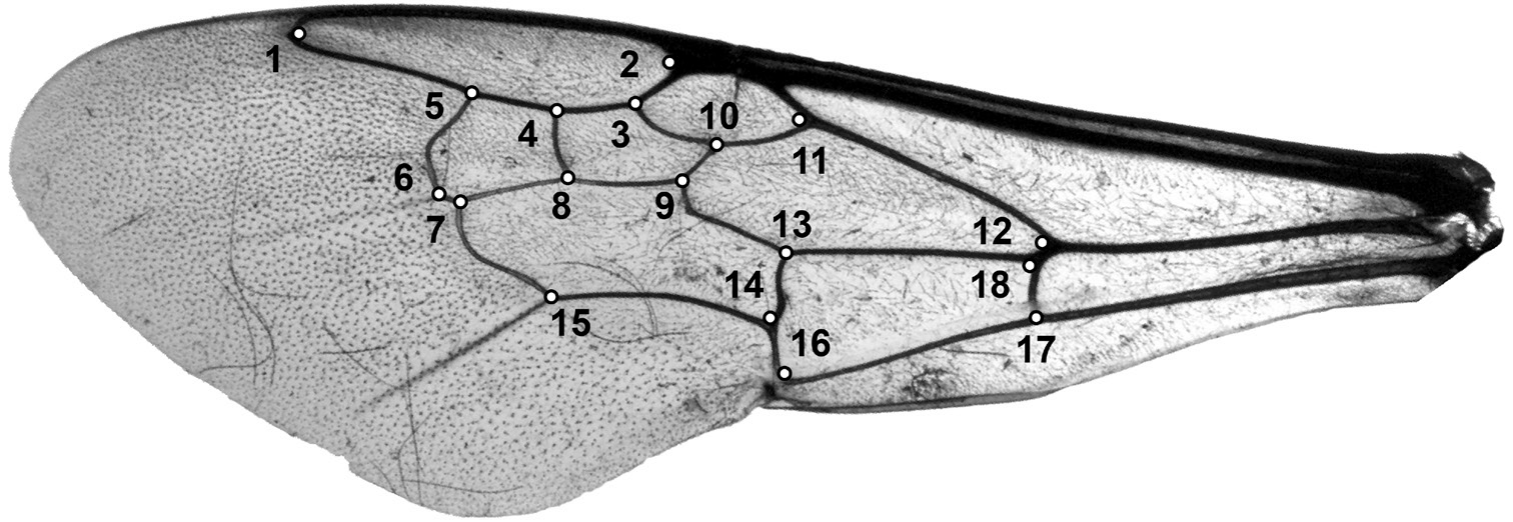
Left forewing of Bombus (Bombus) terrestris (Linnaeus, 1758) with the 18 landmark points indicated on the veins to describe the shape (photograph by Michaël Terzo). The names of the veins and cells can be found in Dehon et al. (2017).

### Taxonomic affinities of *Calyptapis florissantensis* and *Oligobombus cuspidatus*

When using the first dataset with tribe a priori grouping, the most similar tribe to *C. florissantensis* (i.e., both specimens) and *O. cuspidatus* is Electrapini, while Tetrapediini is the second most similar tribe to *C. florissantensis* and Tetrapediini is the second most similar tribe to *O. cuspidatus* (LDA 3; Suppl material 10: Table S10). When using the second dataset, the most similar tribe to the holotype of *C. florissantensis* is Bombini, while the most similar tribe to the specimen described by Cockerell (1908) is Electrapini (the second most similar tribe being Bombini). The most similar tribe to *O. cuspidatus* is Bombini when using the second dataset (LDA 4; Suppl material 12: Table S12). Those results might be explained by the fact that when using the first dataset, only 20 specimens (i.e., four species, with five specimens per species) were chosen to represent Bombini, compared to 841 specimens (i.e., 210 species) in the second dataset and thereby more fully encompasses the breadth of morphospace represented among modern bombines. Furthermore, the species used to represent Bombini in the first dataset do not represent early-branching subgenera, which were found to be the most similar subgenera to *C. florissantensis* and *O. cuspidatus* in the third dataset (i.e., *Bombias*) (LDA 5; Suppl material 13: Table S13). The similarity of *O. cuspidatus* with Electrapini when using the first dataset is concordant with Antropov et al. (2014), who considered it as a possible member of Bombini with mixed features of Bombini and other corbiculate tribes (i.e., the extinct Electrapini, Electrobombini, Melikertini, and the extant Euglossini). This fossil may represent an extinct stem-group to Bombini and it would explain the different results obtained with the different datasets; those similarities across Bombini and the other tribes representing symplesiomorphic features encompassing the clade. Based on morphological features (i.e., presence of a corbicula, the forewing venation similar to *Bombus* s. l.) and forewing shape similarities, *C. florissantensis* could also belong to a stem-group bombine (Cockerell 1906, 1908; Zeuner and Manning 1976). Based on the available evidence, the conservative position is to consider both species as possible stem-group Bombini. It would be highly desirable to verify this hypothesis using cladistic analyses of new morphological characters in the future.

### Origin and diversification of bumble bees

**Figure 5.**
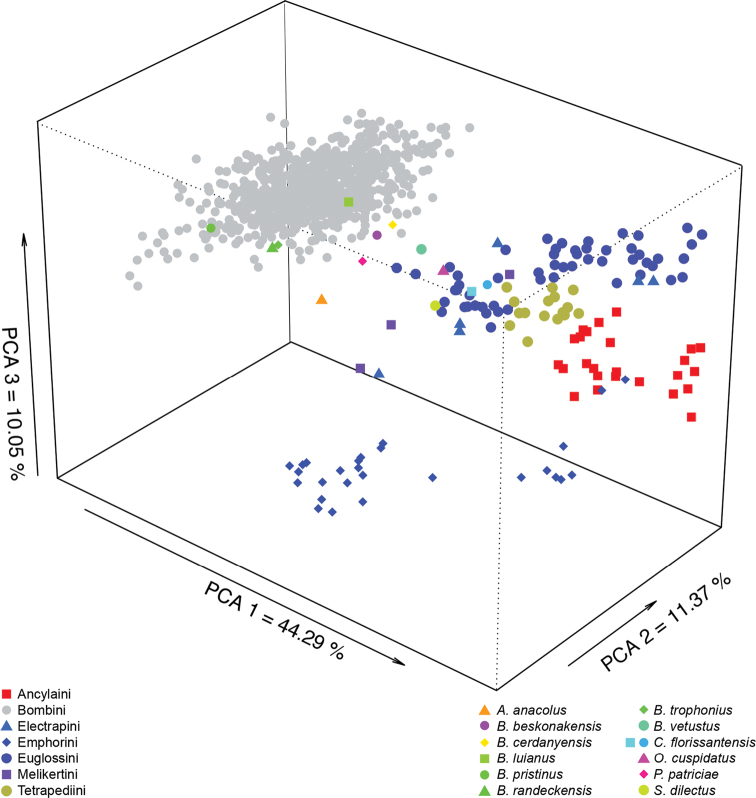
Ordination of the fossils along the three first axes of the PCA (PC1 = 44.29%, PC2 = 11.37%, PCA3 = 10.05%) in subgeneric dataset of *Bombus* s. l.

Our results generally support the timing of divergence of extant species proposed by Williams (1985) and Hines (2008) (Fig. 5), noting that the meager record available for Bombini means such corroboration is minimal at best, albeit non-contradictory. The record of fossil bumble bees is sufficiently scant that at its best we can conclude that the available record does not contradict prior estimates, and falls in line with those for the subgenera *Cullumanobombus*, *Melanobombus*, and *Mendacibombus*. Unfortunately, fossils of most lineages within *Bombus* and certainly from more numerous and refined slices of time are simple lacking, meaning that the current record of fossil bumble bees lacks resolution for determining the timing of most diversification events (e.g., most fossils are clustered within a few deposits representing widely disparate slices in the Oligocene and Miocene). Nonetheless, none of the specimens from Eocene and Oligocene deposits were assigned within the shape space of any contemporary subgenus of *Bombus*, which is not surprising when looking at more completely preserved bees from, for example, the Eocene amber deposits (Baltic, Cambay, Rovno) (e.g., Engel 2001). On the other hand, most specimens coming from Miocene deposits were assigned within the contemporary shape space of *Bombus* s. l., and for some of them within contemporary subgenera (i.e., *Cullumanobombus*, *Melanobombus*, and *Mendacibombus*), again a pattern consistent with more completely preserved bees from other Miocene deposits (e.g., Dominican, Mexican amber) (e.g., Engel et al. 2012). This pattern mirrors the hypothesis that there were significant changes in the bee fauna between Eocene and Oligocene epochs and again at the Paleogene-Neogene transition (e.g., Engel 2001, 2004, 2019a, b). Contemporary species of *Melanobombus* and *Mendacibombus* are restricted to the Old World. On the other hand, most species of *Cullumanobombus* (excluding *B. cullumanus*, *B. semenoviellus*, *B. unicus*) occur in the New World (Williams 1998; Williams et al. 2014). *Cullumanobombus* and *Melanobombus* are estimated to have originated between 20.0–15.0 Ma, while the basal subgenus Mendacibombus has been estimated to have originated around 34.0–30.0 Ma (Hines 2008). All fossils having affinities to extant subgenera have an age that is posterior to the stem age of subgenera (Hines 2008), and therefore our assignments are coincident with the estimated origin and divergence times of *Bombus* s. l. and extant subgenera (Hines 2008).

Other fossil specimens that were assigned to extant subgenera of *Bombus* s. l. are in accordance with the estimated stem age of those groups. In occurrence, our analyses are concordant with the ages of *Cullumanobombus* and *Melanobombus* (i.e., between 20.00–15.00 Ma), as well as *Mendacibombus* (i.e., between 34.0–30.0 Ma) (Hines 2008). These species highlight that Bombini had diversified significantly by the Miocene and that these limited fossil data are concordant with dating estimates. Continued paleontological exploration will only further refine our understanding based on direct evidence for dating bumble bee evolution.

### Cenozoic extinctions of Corbiculata

Corbiculata are the most represented bees in the fossil record, especially in terms of number of specimens found in amber deposits, with workers of certain stingless bees (Meliponini) numbering into the tens of thousands of individuals (Michez et al. 2012; Engel and Michener 2013b; Engel pers. obs.). Corbiculata appeared in the Late Cretaceous based on the occurrence of a crown-group meliponine in Maastrichtian-aged Raritan amber (Michener and Grimaldi 1988; Engel 2000). This indicates that the divergence events among the tribes, at least among their stem groups, extend back to at least the latest Cretaceous. The three extinct tribes of corbiculate bees Electrapini, Electrobombini, and Melikertini are known from the Eocene (Baltic amber, Cambay amber, various impression fossil deposits such as Messel and Eckfeld), and some of these were assuredly advanced eusocial like Apini and Meliponini based on the presence of morphologically specialized workers (Engel 2001). Dehon et al. (2014) described a new corbiculate species, *Euglossopteryx biesmeijeri* De Meulemeester, Michez & Engel, 2014 discovered in the Parachute Creek Member of the Green River Formation (Utah, USA), and had phenetic similarities in wing shape to Euglossini, but it remains to be determined whether this was symplesiomorphic similarity or indicative of a cladistic relationship.

During the Paleocene-Eocene (the Paleocene-Eocene Thermal Maximum and Eocene Thermal Optimum), the concentration of greenhouse gases and the mean global temperature was higher than at present, with poles with little to no ice (Zachos et al. 2001; Royer 2006). The Early Eocene was marked by the EECO (Early Eocene Climatic Optimum) 51–53 million years ago, with a high pCO_2_ and the global temperature reaching a long-term maximum. This was likely caused in part by differences in volcanic emissions, particularly high during parts of the Paleocene-Eocene periods (i.e., 40.0–60.0 Ma) (Walker et al. 1981). The PETM (Paleocene Eocene Thermal Maximum, i.e., 55.0 Ma) is the most prominent and best-studied hyperthermal episode, during which the global temperature increased by more than 5°C in less than 10,000 years (Zachos et al. 2001, 2008). A global cooling that most certainly caused the large-scale extinction of many plant and animal species marked the Eocene-Oligocene transition (i.e., 34.0 Ma) (Zachos et al. 2008; Hansen et al. 2013). Although still speculative at this time, it could be hypothesized that the latter event was related to the extinction of the group of Bombini to which *Calyptapis florissantensis* and *Oligobombus cuspidatus* might have belonged. Similarly, Dehon et al. (2014) suggested that *E. biesmeijeri* could also be consistent with the hypothesis that global climates, particularly cooling and drying events, were somehow related to the loss of corbiculate diversity (Engel 2001, 2002; Engel et al. 2013a), and that this was perhaps a global phenomenon impacting similar bee lineages in the New World (Dehon et al. 2014). It is noticeable that extant bumble bees appear especially sensitive to hyperthermal crises (Kerr et al. 2015; Rasmont et al. 2015). These climatic events resulted in significantly floral turnovers and these florist changes certainly could have influenced ancient lineages of bees (Collinson 1992). Those events of successive changes in temperature might have played a role in the appearance or extinction of species studied herein.

**Figure 6.**
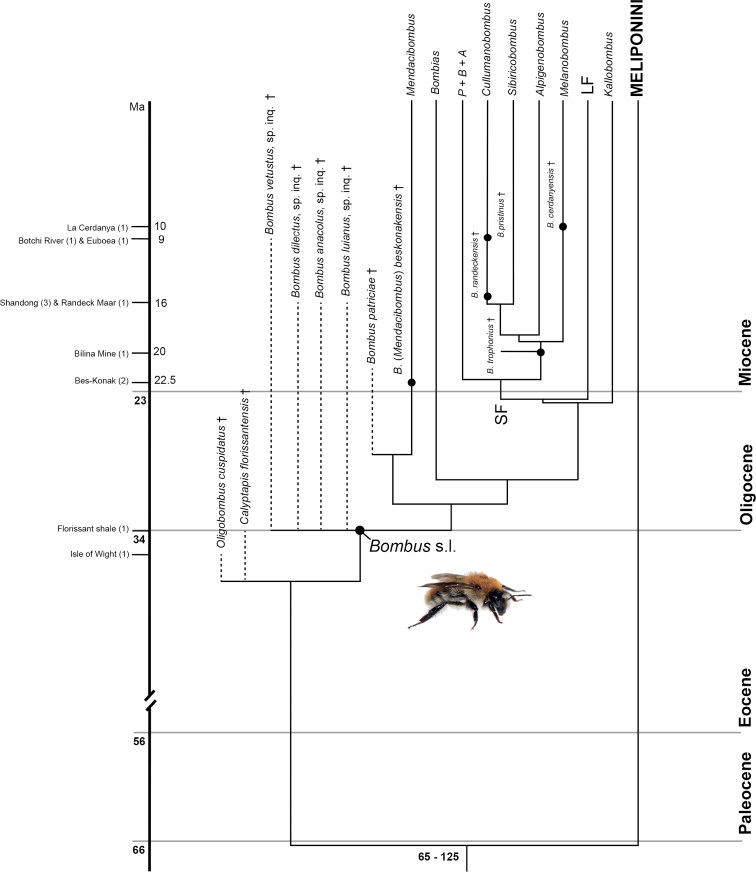
Hypothesis of bumble bee evolution according to the branching dates of Hines (2008) alone with the subgeneric system of Williams et al. (2008). Fossils are mapped onto the clade according to our hypotheses based on our wing morphometry/shape results. Geometric morphometric analyses should be considered as a heuristic tool given the absence of other forms of pertinent data (e.g., absence of information on mandibular form, pretarsal structure, genitalic characters, etc.). A = *Alpinobombus*. B = *Bombus* s.str. LF = clade with mostly long-faced species. P = *Pyrobombus*. SF = clade with mostly short-faced species.

## Supplementary Material

XML Treatment for Oligobombus

XML Treatment for Oligobombus cuspidatus

XML Treatment for Calyptapis

XML Treatment for Calyptapis florissantensis

XML Treatment for Subgenus Paraelectrobombus

XML Treatment for Bombus (Paraelectrobombus) patriciae

XML Treatment for Bombus (Mendacibombus) beskonakensis

XML Treatment for Bombus (Cullumanobombus) trophonius

XML Treatment for Bombus (Cullumanobombus) randeckensis

XML Treatment for “Bombus” luianus

XML Treatment for “Bombus” dilectus

XML Treatment for “Bombus” anacolus

XML Treatment for “Bombus” vetustus

XML Treatment for Bombus (Cullumanobombus) pristinus

XML Treatment for Bombus (Melanobombus) cerdanyensis

## Supplementary materials

Supplementary material 1 Table S1. First dataset for the geometric morphometric analyses Data type: species data Explanation note: This sampling includes 988 specimens from 233 species, 141 genera, 53 tribes, 19 subfamilies, and 7 families of ApoideaAnthophila. N1 = number of species. N2 = number of specimens. https://binary.pensoft.net/file/358119This dataset is made available under the Open Database License (http://opendatacommons.org/licenses/odbl/1.0/). The Open Database License (ODbL) is a license agreement intended to allow users to freely share, modify, and use this Dataset while maintaining this same freedom for others, provided that the original source and author(s) are credited.Manuel Dehon, Michael S. Engel, Maxence Gérard, A. Murat Aytekin, Guillaume Ghisbain, Paul H. Williams, Pierre Rasmont, Denis Michez

Supplementary material 2 Table S2. Second dataset for the geometric morphometric analyses Data type: species data Explanation note: This sampling includes 973 specimens from 252 species, 19 genera, and five tribes of Apidae. N = number of specimens. https://binary.pensoft.net/file/358120This dataset is made available under the Open Database License (http://opendatacommons.org/licenses/odbl/1.0/). The Open Database License (ODbL) is a license agreement intended to allow users to freely share, modify, and use this Dataset while maintaining this same freedom for others, provided that the original source and author(s) are credited.Manuel Dehon, Michael S. Engel, Maxence Gérard, A. Murat Aytekin, Guillaume Ghisbain, Paul H. Williams, Pierre Rasmont, Denis Michez

Supplementary material 3 Table S3. Specimen assignment in families using the cross-validation procedure in the LDA of forewing shape in first dataset Data type: statistical data Explanation note: Original groups are along the rows, predicted groups are along the columns. The hit ratio (HR%) is given for each family. https://binary.pensoft.net/file/358121This dataset is made available under the Open Database License (http://opendatacommons.org/licenses/odbl/1.0/). The Open Database License (ODbL) is a license agreement intended to allow users to freely share, modify, and use this Dataset while maintaining this same freedom for others, provided that the original source and author(s) are credited.Manuel Dehon, Michael S. Engel, Maxence Gérard, A. Murat Aytekin, Guillaume Ghisbain, Paul H. Williams, Pierre Rasmont, Denis Michez

Supplementary material 4 Table S4. Specimen assignment in subfamilies using the cross-validation procedure in the LDA of forewing shape in first dataset Data type: statistical data Explanation note: Original groups are along the rows, predicted groups are along the columns. The hit ratio (HR%) is given for each subfamily. https://binary.pensoft.net/file/358122This dataset is made available under the Open Database License (http://opendatacommons.org/licenses/odbl/1.0/). The Open Database License (ODbL) is a license agreement intended to allow users to freely share, modify, and use this Dataset while maintaining this same freedom for others, provided that the original source and author(s) are credited.Manuel Dehon, Michael S. Engel, Maxence Gérard, A. Murat Aytekin, Guillaume Ghisbain, Paul H. Williams, Pierre Rasmont, Denis Michez

Supplementary material 5 Table S5. Specimen assignment in tribes using the cross-validation procedure in the LDA of forewing shape in first dataset Data type: statistical data Explanation note: Original groups are along the rows, predicted groups are along the columns. The hit ratio (HR%) is given for each tribe. https://binary.pensoft.net/file/358123This dataset is made available under the Open Database License (http://opendatacommons.org/licenses/odbl/1.0/). The Open Database License (ODbL) is a license agreement intended to allow users to freely share, modify, and use this Dataset while maintaining this same freedom for others, provided that the original source and author(s) are credited.Manuel Dehon, Michael S. Engel, Maxence Gérard, A. Murat Aytekin, Guillaume Ghisbain, Paul H. Williams, Pierre Rasmont, Denis Michez

Supplementary material 6 Table S6. Specimen assignment in tribes using the cross-validation procedure in the LDA of forewing shape in the second dataset Data type: statistical data Explanation note: Original groups are along the rows, predicted groups are along the columns. The hit ratio (HR%) is given for each tribe. https://binary.pensoft.net/file/358124This dataset is made available under the Open Database License (http://opendatacommons.org/licenses/odbl/1.0/). The Open Database License (ODbL) is a license agreement intended to allow users to freely share, modify, and use this Dataset while maintaining this same freedom for others, provided that the original source and author(s) are credited.Manuel Dehon, Michael S. Engel, Maxence Gérard, A. Murat Aytekin, Guillaume Ghisbain, Paul H. Williams, Pierre Rasmont, Denis Michez

Supplementary material 7 Table S7. Specimen assignment in subgenera using the cross-validation procedure in the LDA of forewing shape in the third dataset Data type: statistical data Explanation note: Original groups are along the rows, predicted groups are along the columns. The hit ratio (HR%) is given for each subgenus. https://binary.pensoft.net/file/358125This dataset is made available under the Open Database License (http://opendatacommons.org/licenses/odbl/1.0/). The Open Database License (ODbL) is a license agreement intended to allow users to freely share, modify, and use this Dataset while maintaining this same freedom for others, provided that the original source and author(s) are credited.Manuel Dehon, Michael S. Engel, Maxence Gérard, A. Murat Aytekin, Guillaume Ghisbain, Paul H. Williams, Pierre Rasmont, Denis Michez

Supplementary material 8 Table S8. Specimen assignment in subgenera using the cross-validation procedure in the LDA of forewing shape based on male wing shapes Data type: statistical data Explanation note: Original groups are along the rows, predicted groups are along the columns. The hit ratio (HR%) is given for each family. https://binary.pensoft.net/file/358126This dataset is made available under the Open Database License (http://opendatacommons.org/licenses/odbl/1.0/). The Open Database License (ODbL) is a license agreement intended to allow users to freely share, modify, and use this Dataset while maintaining this same freedom for others, provided that the original source and author(s) are credited.Manuel Dehon, Michael S. Engel, Maxence Gérard, A. Murat Aytekin, Guillaume Ghisbain, Paul H. Williams, Pierre Rasmont, Denis Michez

Supplementary material 9 Table S9. Mahalanobis distances (MD) between family centroids and the 988 specimens, and the fossil and family centroids in the first dataset Data type: statistical data https://binary.pensoft.net/file/358127This dataset is made available under the Open Database License (http://opendatacommons.org/licenses/odbl/1.0/). The Open Database License (ODbL) is a license agreement intended to allow users to freely share, modify, and use this Dataset while maintaining this same freedom for others, provided that the original source and author(s) are credited.Manuel Dehon, Michael S. Engel, Maxence Gérard, A. Murat Aytekin, Guillaume Ghisbain, Paul H. Williams, Pierre Rasmont, Denis Michez

Supplementary material 10 Table S10. Mahalanobis distances (MD) between subfamily centroids and the 988 specimens, and the fossil and subfamily centroids in the first dataset Data type: statistical data https://binary.pensoft.net/file/358128This dataset is made available under the Open Database License (http://opendatacommons.org/licenses/odbl/1.0/). The Open Database License (ODbL) is a license agreement intended to allow users to freely share, modify, and use this Dataset while maintaining this same freedom for others, provided that the original source and author(s) are credited.Manuel Dehon, Michael S. Engel, Maxence Gérard, A. Murat Aytekin, Guillaume Ghisbain, Paul H. Williams, Pierre Rasmont, Denis Michez

Supplementary material 11 Table S11. Mahalanobis distances (MD) between tribe centroids and the 988 specimens, and the fossil and tribe centroids in the first dataset Data type: statistical data https://binary.pensoft.net/file/358129This dataset is made available under the Open Database License (http://opendatacommons.org/licenses/odbl/1.0/). The Open Database License (ODbL) is a license agreement intended to allow users to freely share, modify, and use this Dataset while maintaining this same freedom for others, provided that the original source and author(s) are credited.Manuel Dehon, Michael S. Engel, Maxence Gérard, A. Murat Aytekin, Guillaume Ghisbain, Paul H. Williams, Pierre Rasmont, Denis Michez

Supplementary material 12 Table S12. Mahalanobis distances (MD) between tribe centroids and the 973 specimens, and the fossil and tribe centroids in the second dataset Data type: statistical data https://binary.pensoft.net/file/358130This dataset is made available under the Open Database License (http://opendatacommons.org/licenses/odbl/1.0/). The Open Database License (ODbL) is a license agreement intended to allow users to freely share, modify, and use this Dataset while maintaining this same freedom for others, provided that the original source and author(s) are credited.Manuel Dehon, Michael S. Engel, Maxence Gérard, A. Murat Aytekin, Guillaume Ghisbain, Paul H. Williams, Pierre Rasmont, Denis Michez

Supplementary material 13 Table S13. Mahalanobis distances (MD) between subgenus centroids and the 841 specimens, and the fossils and subgenus centroids in the third dataset Data type: statistical data https://binary.pensoft.net/file/358131This dataset is made available under the Open Database License (http://opendatacommons.org/licenses/odbl/1.0/). The Open Database License (ODbL) is a license agreement intended to allow users to freely share, modify, and use this Dataset while maintaining this same freedom for others, provided that the original source and author(s) are credited.Manuel Dehon, Michael S. Engel, Maxence Gérard, A. Murat Aytekin, Guillaume Ghisbain, Paul H. Williams, Pierre Rasmont, Denis Michez
