# Supplementary material for: Morphometric analysis of fossil bumble bees (Hymenoptera, Apidae, Bombini) reveals their taxonomic affinities
Source: Zookeys. 2019 Nov 21;891:71–118. doi: 10.3897/zookeys.891.36027 (PMC6882928; doi:10.3897/zookeys.891.36027)
Supplement: Supplementary material 1 [file zookeys-891-071-s001.docx]

**Appendix 1 Table S1.** First dataset for the geometric morphometric analyses. This sampling includes 979 specimens from 226 species, 135 genera, 50 tribes, 18 subfamilies, and 7 families of Apoidea Anthophila. N1= number of species. N2 = number of specimens.

| **Family** | **Subfamily / Clade** | **Tribe** | **N1** | **N2** |
| --- | --- | --- | --- | --- |
| Andrenidae | Andreninae | Andrenini | 4 | 20 |
|  |  | Euherbstiini | 3 | 20 |
|  | Oxaeinae |  | 4 | 20 |
|  | Panurginae | Melitturgini | 4 | 20 |
|  |  | Nolanomelissini | 1 | 10 |
|  |  | Protandrenini | 5 | 20 |
| Apidae | “Non-parasitic Apidae” | Ancylaini | 7 | 20 |
|  |  | Apini | 4 | 20 |
|  |  | Bombini | 4 | 20 |
|  |  | Electrapini† | 5 | 6 |
|  |  | Centridini | 4 | 20 |
|  |  | Emphorini | 4 | 20 |
|  |  | Eucerini | 4 | 20 |
|  |  | Euglossini | 4 | 20 |
|  |  | Exomalopsini | 6 | 20 |
|  |  | Melikertini† | 3 | 3 |
|  |  | Tapinotaspidini | 5 | 20 |
|  |  | Tetrapediini | 5 | 20 |
|  | “Parasitic Apidae” | Anthophorini | 6 | 20 |
|  |  | Brachynomadini | 5 | 20 |
|  |  | Ericrocridini | 4 | 20 |
|  |  | Epeolini | 7 | 20 |
|  |  | Isepeolini | 4 | 20 |
|  |  | Melectini | 6 | 20 |
|  |  | Nomadini | 4 | 20 |
|  |  | Osirini | 5 | 20 |
|  |  | Protepeolini | 4 | 20 |
|  |  | Rhathymini | 3 | 20 |
|  | Xylocopinae | Ceratinini | 4 | 20 |
|  |  | Manuelinii | 3 | 20 |
|  |  | Xylocopini | 4 | 20 |
| Colletidae | Callomelittinae |  | 3 | 20 |
|  | Colletinae | Colletini | 4 | 20 |
|  | Neopasiphaeinae |  | 4 | 20 |
|  | Diphaglossinae | Caupolicanini | 7 | 20 |
|  |  | Diphaglossini | 3 | 20 |
|  |  | Dissoglotini | 3 | 20 |
| Halictidae | Halictinae | Augochlorini | 4 | 20 |
|  |  | Caenohalictini | 5 | 20 |
|  |  | Halictini | 5 | 20 |
|  |  | Sphecodini | 5 | 20 |
|  |  | Thrinchostomatini | 4 | 20 |
|  | Nomiinae |  | 4 | 20 |
|  | Nomioidinae |  | 5 | 20 |
|  | Rophitinae | Conanhalictini | 6 | 20 |
|  |  | Penapini | 4 | 19 |
|  |  | Rophitini | 6 | 20 |
|  |  | Xeralictini | 3 | 20 |
| Megachilidae | Fideliinae | Fideliini | 3 | 20 |
| Melittidae | Meganomiinae |  | 6 | 20 |
|  | Melittinae | Melittini | 6 | 20 |
| Stenotritidae |  |  | 8 | 19 |
