# Supplementary material for: Morphometric analysis of fossil bumble bees (Hymenoptera, Apidae, Bombini) reveals their taxonomic affinities
Source: Zookeys. 2019 Nov 21;891:71–118. doi: 10.3897/zookeys.891.36027 (PMC6882928; doi:10.3897/zookeys.891.36027)
Supplement: Supplementary material 2 [file zookeys-891-071-s002.docx]

**Appendix 2 Table S2.** Second dataset for the geometric morphometric analyses. This sampling includes 973 specimens from 252 species, 19 genera, and 5 tribes of Apidae. N = number of specimens

| **Tribe** | **Genus** | **Species** | **N** |
| --- | --- | --- | --- |
| Bombini | *Bombus* Latreille, 1802 | *B.* (*Alpigenobombus*) *breviceps* Smith, 1852 | 5 |
|  |  | *B.* (*Alpigenobombus*) *genalis* Friese, 1918 | 5 |
|  |  | *B.* (*Alpigenobombus*) *kashmirensis* Friese, 1909 | 5 |
|  |  | *B.* (*Alpigenobombus*) *nobilis* Friese, 1905 | 5 |
|  |  | *B.* (*Alpigenobombus) wurlfenii* Radoszkowski, 1860 | 5 |
|  |  | *B.* (*Alpinobombus*) *alpinus* (Linnaeus, 1758) | 3 |
|  |  | *B.* (*Alpinobombu*s) *balteatus* Dahlbom, 1832 | 4 |
|  |  | *B.* (*Alpinobombus*) *hyperboreus* Schönherr, 1809 | 5 |
|  |  | *B.* (*Alpinobombus*) *neoboreus* Sladen, 1919 | 3 |
|  |  | *B.* (*Alpinobombus*) *polaris* Curtis, 1835 | 5 |
|  |  | *B.* (*Bombias*) *auricomus* (Robertson, 1903) | 5 |
|  |  | *B.* (*Bombias*) *confusus* Schenck, 1861 | 5 |
|  |  | *B.* (*Bombias*) *nevadensis* Cresson, 1874 | 5 |
|  |  | *B.* (*Bombus*) *affinis* Cresson, 1863 | 1 |
|  |  | *B.* (*Bombus*) *cryptarum* (Fabricius, 1775) | 5 |
|  |  | *B.* (*Bombus*) *hypocrita* Pérez, 1905 | 2 |
|  |  | *B.* (*Bombus*) *lucorum* (Linnaeus, 1761) | 1 |
|  |  | *B.* (*Bombus*) *magnus* Vogt, 1911 | 2 |
|  |  | *B.* (*Bombus*) *moderatus* (Linnaeus, 1761) | 1 |
|  |  | *B.* (*Bombus*) *patagiatus* Nylander, 1848 | 4 |
|  |  | *B.* (*Bombus*) *sporadicus* Nylander, 1848 | 3 |
|  |  | *B.* (*Bombus*) *terrestris* (Linnaeus, 1758) | 5 |
|  |  | *B.* (*Bombus*) *terricola* Kirby, 1837 | 2 |
|  |  | *B.* (*Bombus*) *tunicatus* Smith, 1852 | 1 |
|  |  | *B.* (*Cullumanobombus*) *baeri* Vachal, 1904 | 5 |
|  |  | *B.* (*Cullumanobombus*) *brachycephalus* Handlirsch, 1888 | 5 |
|  |  | *B.* (*Cullumanobombus*) *coccineus* Friese, 1903 | 3 |
|  |  | *B.* (*Cullumanobombus*) *crotchii* Cresson, 1878 | 5 |
|  |  | *B.* (*Cullumanobombus*) *cullumanus* (Kirby, 1802) | 3 |
|  |  | *B.* (*Cullumanobombus*) *ecuadorius* Meunier, 1890 | 5 |
|  |  | *B.* (*Cullumanobombus*) *fraternus* (Smith, 1854) | 5 |
|  |  | *B.* (*Cullumanobombus*) *funebris* Smith, 1854 | 5 |
|  |  | *B.* (*Cullumanobombus*) *griseocollis* (DeGeer, 1773) | 5 |
|  |  | *B.* (*Cullumanobombus*) *handlirschi* Friese, 1903 | 5 |
|  |  | *B.* (*Cullumanobombus*) *haueri* Handlirsch, 1888 | 5 |
|  |  | *B.* (*Cullumanobombus*) *hortunalus* Friese, 1904 | 5 |
|  |  | *B.* (*Cullumanobombus*) *macgregori* LaBougle & Ayala, 1985 | 1 |
|  |  | *B.* (*Cullumanobombus*) *melaleucus* Handlirsch, 1888 | 5 |
|  |  | *B.* (*Cullumanobombus*) *morrisoni* Cresson, 1878 | 5 |
|  |  | *B.* (*Cullumanobombus*) *robustus* Smith, 1854 | 5 |
|  |  | *B.* (*Cullumanobombus*) *rubincundus* Smith, 1854 | 5 |
|  |  | *B.* (*Cullumanobombus*) *rufocinctus* Cresson, 1863 | 5 |
|  |  | *B.* (*Cullumanobombus*) *tucumanus* Vachal, 1904 | 5 |
|  |  | *B.* (*Cullumanobombus*) *unicus* Morawitz, 1883 | 1 |
|  |  | *B.* (*Cullumanobombus*) *vogti* Friese, 1903 | 5 |
|  |  | *B.* (*Cullumanobombus*) *volucelloides* Gribodo, 1892 | 5 |
|  |  | *B.* (*Kallobombus*) *soroeensis* (Fabricius, 1776) | 5 |
|  |  | *B.* (*Megabombus*) *argillaceus* (Scopoli, 1763) | 5 |
|  |  | *B.* (*Megabombus*) *bicoloratus* Smith, 1879 | 4 |
|  |  | *B.* (*Megabombus*) *consobrinus* Dahlbom, 1832 | 3 |
|  |  | *B.* (*Megabombus*) *czerksii* Skorikov, 1910 | 2 |
|  |  | *B.* (*Megabombus*) *diversus* Smith, 1869 | 5 |
|  |  | *B.* (*Megabombus*) *hortorum* (Linnaeus, 1761) | 5 |
|  |  | *B.* (*Megabombus*) *koreanus* (Skorikov, 1933) | 4 |
|  |  | *B.* (*Megabombus*) *kulingensis* Cockerell, 1917 | 2 |
|  |  | *B.* (*Megabombus*) *longipes* Friese, 1905 | 2 |
|  |  | *B.* (*Megabombus*) *portchinsky* Radoszkowski, 1883 | 4 |
|  |  | *B.* (*Megabombus*) *religiosus* (Frison, 1935) | 2 |
|  |  | *B.* (*Megabombus*) *securus* (Frison, 1935) | 1 |
|  |  | *B.* (*Megabombus*) *senex* Vollenhoven, 1873 | 5 |
|  |  | *B.* (*Megabombus*) *supremus* Morawitz, 1887 | 1 |
|  |  | *B.* (*Megabombus*) *sushkini* (Skorikov, 1931) | 4 |
|  |  | *B.* (*Megabombus*) *trifasciatus* Smith, 1852 | 5 |
|  |  | *B.* (*Megabombus*) *ussuriensis* Radoszkowski, 1877 | 5 |
|  |  | *B.* (*Melanobombus*) *erzurumensis* (Özbek, 1990) | 2 |
|  |  | *B.* (*Melanobombus*) *eximius* Smith, 1852 | 5 |
|  |  | *B.* (*Melanobombus*) *festivus* Smith, 1861 | 5 |
|  |  | *B.* (*Melanobombus*) *friseanus* Skorikov, 1933 | 5 |
|  |  | *B.* (*Melanobombus*) *incertus* Morawitz, 1881 | 5 |
|  |  | *B.* (*Melanobombus*) *keriensis* Morawitz, 1887 | 5 |
|  |  | *B.* (*Melanobombus*) *ladakhensis* Richards, 1928 | 5 |
|  |  | *B.* (*Melanobombus*) *lapidarius* (Linnaeus, 1758) | 5 |
|  |  | *B.* (*Melanobombus*) *miniatus* Bingham, 1897 | 3 |
|  |  | *B.* (*Melanobombus*) *pyrosoma* Morawitz, 1890 | 5 |
|  |  | *B.* (*Melanobombus*) *richardsiellus* (Tkalců, 1968) | 1 |
|  |  | *B.* (*Melanobombus*) *rufipes* Lepeletier, 1836 | 5 |
|  |  | *B.* (*Melanobombus*) *rufofasciatus* Smith, 1852 | 5 |
|  |  | *B.* (*Melanobombus*) *semenovianus* (Skorikov, 1914) | 1 |
|  |  | *B.* (*Melanobombus*) *sichelii* Radoszkowski, 1869 | 2 |
|  |  | *B.* (*Melanobombus*) *simillimus* Smith, 1852 | 5 |
|  |  | *B.* (*Melanobombus*) *tanguticus* Morawitz, 1887 | 4 |
|  |  | *B.* (*Mendacibombus*) *avinoviellus* (Skorikov, 1914) | 3 |
|  |  | *B.* (*Mendacibombus*) *defector* Skorikov, 1910 | 1 |
|  |  | *B.* (*Mendacibombus*) *handlirschianus* Vogt, 1909 | 3 |
|  |  | *B.* (*Mendacibombus*) *himalayanus* (Skorikov, 1914) | 1 |
|  |  | *B.* (*Mendacibombus*) *marussinus* Skorikov, 1910 | 2 |
|  |  | *B.* (*Mendacibombus*) *mendax* Gerstäcker, 1869 | 5 |
|  |  | *B.* (*Mendacibombus*) *shaposhnikovi* Skorikov, 1910 | 1 |
|  |  | *B.* (*Mendacibombus*) *turkestanicus* Skorikov, 1910 | 1 |
|  |  | *B.* (*Mendacibombus*) *waltoni* Cockerell, 1910 | 2 |
|  |  | *B.*(*Orientalibombus*) *funerarius* Smith, 1852 | 5 |
|  |  | *B.*(*Orientalibombus*) *haemorrhoidalis* Smith, 1852 | 5 |
|  |  | *B.* (*Psithyrus*) *ashtoni* (Cresson, 1864) | 5 |
|  |  | *B.* (*Psithyrus*) *bellardii* (Gribodo, 1892) | 3 |
|  |  | *B.* (*Psithyrus*) *branickii* (Radoszkowski, 1893) | 5 |
|  |  | *B.* (*Psithyrus*) *campestris* (Panzer, 1801) | 1 |
|  |  | *B.* (*Psithyrus*) *citrinus* (Smith, 1854) | 5 |
|  |  | *B.* (*Psithyrus*) *ferganicus* (Radoszkowski, 1893) | 5 |
|  |  | *B.* (*Psithyrus*) *fernaldae* (Franklin, 1911) | 5 |
|  |  | *B.* (*Psithyrus*) *insularis* (Smith, 1861) | 5 |
|  |  | *B.* (*Psithyrus*) *intrudens* (Smith, 1861) | 5 |
|  |  | *B.* (*Psithyrus*) *morawitzianus* (Popov, 1931) | 1 |
|  |  | *B.* (*Psithyrus*) *skorikovi* (Popov, 1927) | 5 |
|  |  | *B.* (*Psithyrus*) *suckleyi* Greene, 1860 | 5 |
|  |  | *B.* (*Psithyrus*) *variabilis* (Cresson, 1872) | 5 |
|  |  | *B.* (*Pyrobombus*) *abnormis* (Tkalců, 1968) | 1 |
|  |  | *B.* (*Pyrobombus*) *ardens* Smith, 1879 | 5 |
|  |  | *B.* (*Pyrobombus*) *beaticola* (Tkalců, 1968) | 2 |
|  |  | *B.* (*Pyrobombus*) *bifarius* Cresson, 1878 | 5 |
|  |  | *B.* (*Pyrobombus*) *bimaculatus* Cresson, 1863 | 5 |
|  |  | *B.* (*Pyrobombus*) *biroi* Vogt, 1911 | 5 |
|  |  | *B.* (*Pyrobombus*) *caliginosus* (Frison, 1927) | 5 |
|  |  | *B.* (*Pyrobombus*) *centralis* Cresson, 1864 | 5 |
|  |  | *B.* (*Pyrobombus*) *ephippiatus* Say, 1837 | 5 |
|  |  | *B.* (*Pyrobombus*) *flavescens* Smith, 1852 | 5 |
|  |  | *B.* (*Pyrobombus*) *flavifrons* Cresson, 1863 | 5 |
|  |  | *B.* (*Pyrobombus*) *frigidus* Smith, 1854 | 5 |
|  |  | *B.* (*Pyrobombus*) *haematurus* Kriechbaumer, 1870 | 2 |
|  |  | *B.* (*Pyrobombus*) *huntii* Greene, 1860 | 5 |
|  |  | *B.* (*Pyrobobmus*) *hypnorum* (Linnaeus, 1758) | 3 |
|  |  | *B.* (*Pyrobombus*) *impatiens* Cresson, 1863 | 5 |
|  |  | *B.* (*Pyrobombus*) *infrequens* (Tkalců, 1989) | 4 |
|  |  | *B.* (*Pyrobombus*) *lemniscatus* Skorikov, 1912 | 5 |
|  |  | *B.* (*Pyrobombus*) *lepidus* Skorikov, 1912 | 5 |
|  |  | *B.* (*Pyrobombus*) *luteipes* Richards, 1934 | 5 |
|  |  | *B.* (*Pyrobombus*) *melanopygus* Nylander, 1848 | 5 |
|  |  | *B.* (*Pyrobombus*) *mirus* (Tkalců, 1968) | 3 |
|  |  | *B.* (*Pyrobombus*) *mixtus* Cresson, 1878 | 5 |
|  |  | *B.* (*Pyrobombus*) *parthenius* Richards, 1934 | 3 |
|  |  | *B.* (*Pyrobombus*) *perplexus* Cresson, 1863 | 5 |
|  |  | *B.* (*Pyrobombus*) *picipes* Richards, 1934 | 5 |
|  |  | *B.* (*Pyrobombus*) *pleuralis* Nylander, 1848 | 5 |
|  |  | *B.* (*Pyrobombus*) *pratorum* (Linnaeus, 1761) | 3 |
|  |  | *B.* (*Pyrobombus*) *pressus* (Frison, 1935) | 5 |
|  |  | *B.* (*Pyrobombus*) *pullatus* Franklin, 1913 | 5 |
|  |  | *B.* (*Pyrobombus*) *rotundiceps* Friese, 1916 | 3 |
|  |  | *B.* (*Pyrobombus*) *sandersoni* Franklin, 1913 | 5 |
|  |  | *B.* (*Pyrobombus*) *sitkensis* Nylander, 1848 | 5 |
|  |  | *B.* (*Pyrobombus*) *sonani* (Frison, 1934) | 1 |
|  |  | *B.* (*Pyrobombus*) *subtypicus* (Skorikov, 1914) | 5 |
|  |  | *B.* (*Pyrobombus*) *sylvicola* Kirby, 1837 | 5 |
|  |  | *B.* (*Pyrobombus*) *ternarius* Say, 1837 | 5 |
|  |  | *B.* (*Pyrobombus*) *vagans* Smith, 1854 | 5 |
|  |  | *B.* (*Pyrobombus*) *vandykei* (Frison, 1927) | 5 |
|  |  | *B.* (*Pyrobombus*) *vosnesenskii* Radoszkowski, 1862 | 5 |
|  |  | *B.* (*Pyrobombus*) *wilmattae* Cockerell, 1912 | 4 |
|  |  | *B.* (*Sibiricobombus*) *asiaticus* Morawitz, 1875 | 5 |
|  |  | *B.* (*Sibiricobombus*) *morawitzi* Radoszkowski, 1876 | 4 |
|  |  | *B.* (*Sibiricobombus*) *niveatus* Kriechbaumer, 1870 | 5 |
|  |  | *B.* (*Sibiricobombus*) *oberti* Morawitz, 1883 | 1 |
|  |  | *B.* (*Sibiricobombus*) *obtusus* Richards, 1951 | 5 |
|  |  | *B.* (*Sibiricobombus*) *sibiricus* (Fabricius, 1781) | 5 |
|  |  | *B.* (*Sibiricobombus*) *sulfureus* Friese, 1905 | 4 |
|  |  | *B.* (*Subterraenobombus*) *amurensis* Radoszkowski, 1861 | 2 |
|  |  | *B.* (*Subterraenobombus*) *appositus* (Skorikov, 1914) | 5 |
|  |  | *B.* (*Subterraenobombus*) *borealis* Kirby, 1837 | 5 |
|  |  | *B.* (*Subterraenobombus*) *difficillimus* Skorikov, 1912 | 3 |
|  |  | *B.* (*Subterraenobombus*) *distinguendus* Morawitz, 1869 | 3 |
|  |  | *B.* (*Subterraenobombus*) *fedtschenkoi* Morawitz, 1875 | 2 |
|  |  | *B.* (*Subterraenobombus*) *fragrans* (Pallas, 1771) | 4 |
|  |  | *B.* (*Subterraenobombus*) *melanurus* Lepeletier, 1836 | 3 |
|  |  | *B.* (*Subterraenobombus*) *mongolensis* Williams, 2011 | 1 |
|  |  | *B.* (*Subterraenobombus*) *personatus* Smith, 1879 | 5 |
|  |  | *B.* (*Subterraenobombus*) *subterraneus* (Linnaeus, 1758) | 5 |
|  |  | *B.* (*Thoracobombus*) *anachoreta* (Skorikov, 1914) | 1 |
|  |  | *B.* (*Thoracobombus*) *armeniacus* Radoszkowski, 1877 | 5 |
|  |  | *B.* (*Thoracobombus*) *atratus* Franklin, 1913 | 5 |
|  |  | *B.* (*Thoracobombus*) *atripes* Smith, 1852 | 5 |
|  |  | *B.* (*Thoracobombus*) *bellicosus* Smith, 1879 | 5 |
|  |  | *B.* (*Thoracobombus*) *brasiliensis* Lepeletier, 1836 | 5 |
|  |  | *B.* (*Thoracobombus*) *brevivillus* Franklin, 1913 | 5 |
|  |  | *B.* (*Thoracobombus*) *californicus* Smith, 1854 | 5 |
|  |  | *B.* (*Thoracobombus*) *dahlbomii* Guérin-Méneville, 1835 | 5 |
|  |  | *B.* (*Thoracobombus*) *deuteronymus* Schulz, 1906 | 1 |
|  |  | *B.* (*Thoracobombus*) *digressus* (Milliron, 1962) | 1 |
|  |  | *B.* (*Thoracobombus*) *diligens* Smith, 1861 | 5 |
|  |  | *B.* (*Thoracobombus*) *excellens* Smith, 1879 | 5 |
|  |  | *B.* (*Thoracobombus*) *exil* (Skorikov, 1923) | 5 |
|  |  | *B.* (*Thoracobombus*) *fervidus* (Fabricius, 1798) | 5 |
|  |  | *B.* (*Thoracobombus*) *filchnerae* Vogt, 1908 | 5 |
|  |  | *B.* (*Thoracobombus*) *honshuensis* (Tkalců, 1968) | 2 |
|  |  | *B.* (*Thoracobombus*) *humilis* Illiger, 1806 | 5 |
|  |  | *B.* (*Thoracobombus*) *imitator* Pittioni, 1949 | 5 |
|  |  | *B.* (*Thoracobombus*) *inexspectatus* (Tkalců, 1963) | 2 |
|  |  | *B.* (*Thoracobombus*) *laesus* Morawitz, 1875 | 5 |
|  |  | *B.* (*Thoracobombus*) *medius* Cresson, 1863 | 5 |
|  |  | *B.* (*Thoracobombus*) *mesomelas* Gerstäcker, 1869 | 5 |
|  |  | *B.* (*Thoracobombus*) *mexicanus* Cresson, 1878 | 5 |
|  |  | *B.* (*Thoracobombus*) *mlokosievitzii* Radoszkowski, 1877 | 2 |
|  |  | *B.* (*Thoracobombus*) *morio* (Swederus, 1787) | 5 |
|  |  | *B.* (*Thoracobombus*) *mucidus* Gerstäcker, 1869 | 5 |
|  |  | *B.* (*Thoracobombus*) *muscorum* (Linnaeus, 1758) | 2 |
|  |  | *B.* (*Thoracobombus*) *opifex* Smith, 1879 | 5 |
|  |  | *B.* (*Thoracobombus*) *pascuorum* (Scopoli, 1763) | 5 |
|  |  | *B.* (*Thoracobombus*) *pensylvanicus* (DeGeer, 1773) | 5 |
|  |  | *B.* (*Thoracobombus*) *persicus* Radoszkowski, 1881 | 5 |
|  |  | *B.* (*Thoracobombus*) *pomorum* (Panzer, 1805) | 5 |
|  |  | *B.* (*Thoracobombus*) *pseudobaicalensis* Vogt, 1911 | 2 |
|  |  | *B.* (*Thoracobombus*) *ruderarius* (Müller, 1776) | 5 |
|  |  | *B.* (*Thoracobombus*) *schrencki* Morawitz, 1881 | 4 |
|  |  | *B.* (*Thoracobombus*) *sonorus* Say, 1837 | 5 |
|  |  | *B.* (*Thoracobombus*) *steindachneri* Handlirsch, 1888 | 5 |
|  |  | *B.* (*Thoracobombus*) *sylvarum* (Linnaeus, 1761) | 5 |
|  |  | *B.* (*Thoracobombus*) *transversalis* (Olivier, 1789) | 5 |
|  |  | *B.* (*Thoracobombus*) *tricornis* Radoszkowski, 1888 | 5 |
|  |  | *B.* (*Thoracobombus*) *trinomanitus* Dalla Torre, 1890 | 4 |
|  |  | *B.* (*Thoracobombus*) *velox* (Skorikov, 1914) | 3 |
|  |  | *B.* (*Thoracobombus*) *weisi* Friese, 1903 | 5 |
|  |  | *B.* (*Thoracobombus*) *zonatus* Smith, 1854 | 5 |
| Ancylaini | *Ancyla* Lepeletier de Saint Fargeau, 1841 | *A. asiatica* Friese, 1922 | 1 |
|  |  | *A. holtzi* Friese, 1902 | 5 |
|  |  | *A. nigricornis* Friese, 1902 | 1 |
|  |  | *A. orientalica*  Warncke, 1979 | 1 |
|  |  | *A.* sp. | 2 |
|  | *Tarsalia* Morawitz, 1895 | *T. ancyliformis* Popov, 1935 | 7 |
|  |  | *T. hirtipes* Morawitz, 1895 | 6 |
|  |  | *T. persica* (Warncke, 1979) | 2 |
| Electrapini | *Electrapis* Cockerell, 1908 | *E. krishnorum* Engel, 2001 | 1 |
|  |  | *E. meliponoides* (Buttel-Reepen, 1906) | 1 |
|  | *Protobombus* Cockerell, 1908 | *P. basilaris* Engel, 2001 | 1 |
|  |  | *P. hirsutus* (Cockerell, 1908) | 1 |
|  | *Thaumastobombus* Engel, 2001 | *T. andreniformis* Engel, 2001 | 2 |
| Emphorini | *Ancyloscelis* Latreille, 1829 | *A. apiformis* (Fabricius, 1793) | 5 |
|  |  | *A. melanostomus* Cockerell, 1923 | 1 |
|  | *Diadasia* Patton, 1879 | *D. afflicta* (Cresson, 1878) | 1 |
|  |  | *D. australis* (Cresson, 1878) | 4 |
|  |  | *D.* *diminuta* (Cresson, 1878) | 1 |
|  |  | *D.* *ochracea* (Cockerell, 1903) | 1 |
|  |  | *D. olivacea* (Cresson, 1878) | 1 |
|  |  | *D. opuntiae* Cockerell, 1901 | 1 |
|  |  | *D. rincornis* Cockerell, 1897 | 1 |
|  | *Melitoma* Murray, 1867 | *M. segmentaria* (Fabricius, 1804) | 5 |
|  | *Ptilothrix* Smith, 1853 | *P. fructifera* (Holmberg, 1903) | 1 |
|  |  | *P. plumata* Smith, 1853 | 5 |
| Euglossini | *Aglae* Lepeletier & Serville, 1825 | *A. caerula* Lepeletier & Audinet-Serville 1825 | 5 |
|  | *Euglossa* Latreille, 1802 | *E. intersecta* Latreille 1838 | 4 |
|  | *Eufriesea* Cockerell, 1908 | *E. surinamensis* (Linnaeus, 1758) | 5 |
|  |  | *E. violacea* (Blanchard, 1840) | 5 |
|  | *Eulaema* Lepeletier, 1841 | *E. cingulata* (Fabricius, 1804) | 5 |
|  |  | *E. nigrita* Lepeletier, 1841 | 5 |
|  |  | *E.* *meriana* (Olivier, 1789) | 5 |
|  |  | *E. luteola* Moure, 1967 | 5 |
|  | *Exaerete* Hoffmannsegg, 1817 | *E. dentata* (Linnaeus, 1758) | 6 |
|  |  | *E. frontalis* (Guérin-Méneville, 1844) | 5 |
|  |  | *E. smaragdina* (Guérin-Méneville, 1844) | 5 |
| Melikertini | *Melikertes* Engel, 2001 | *M. stilbonotus* (Engel, 2001) | 1 |
|  | *Melissites* Engel, 2001 | *M. trigona* Engel, 2001 | 1 |
|  | *Succinapis* Engel, 2001 | *S. goeleti* Engel, 2011 | 1 |
| Tetrapediini | *Tetrapedia* Klug, 1810 | *T. diversipes* Klug, 1810 | 5 |
|  |  | *T. maura* Cresson, 1878 | 5 |
|  |  | *T. regulosa* (probably) | 5 |
|  |  | *T.* sp. | 1 |
