# Supplementary material for: Morphometric analysis of fossil bumble bees (Hymenoptera, Apidae, Bombini) reveals their taxonomic affinities
Source: Zookeys. 2019 Nov 21;891:71–118. doi: 10.3897/zookeys.891.36027 (PMC6882928; doi:10.3897/zookeys.891.36027)
Supplement: Supplementary material 3 [file zookeys-891-071-s003.docx]

**Appendix 3 Table S3.** Specimen assignment in families using the cross-validation procedure in the LDA of forewing shape in first dataset. Original groups are along the rows, predicted groups are along the columns. The hit ratio (HR%) is given for each family.

| **Defined groups** | **Cross-validation** | | | | | | | |
| --- | --- | --- | --- | --- | --- | --- | --- | --- |
|  | Andrenidae | Apidae | Colletidae | Halictidae | Megachilidae | Melittidae | Stenotritidae | **%** |
| Andrenidae | **101** | 3 | 3 | 2 | 0 | 0 | 0 | **92.66** |
| Apidae | 5 | **451** | 8 | 4 | 0 | 1 | 0 | **96.16** |
| Colletidae | 1 | 0 | **109** | 1 | 0 | 0 | 1 | **97.32** |
| Halictidae | 3 | 0 | 2 | **214** | 0 | 0 | 0 | **97.72** |
| Megachilidae | 0 | 0 | 0 | 0 | **20** | 0 | 0 | **100** |
| Melittidae | 1 | 0 | 0 | 0 | 0 | **34** | 5 | **85.00** |
| Stenotritidae | 0 | 0 | 3 | 0 | 0 | 2 | **14** | **73.68** |
