# Supplementary material for: Morphometric analysis of fossil bumble bees (Hymenoptera, Apidae, Bombini) reveals their taxonomic affinities
Source: Zookeys. 2019 Nov 21;891:71–118. doi: 10.3897/zookeys.891.36027 (PMC6882928; doi:10.3897/zookeys.891.36027)
Supplement: Supplementary material 6 [file zookeys-891-071-s006.docx]

**Appendix 6 Table S6.** Specimen assignment in tribes using the cross-validation procedure in the LDA of forewing shape in the second dataset. Original groups are along the rows, predicted groups are along the columns. The hit ratio (HR%) is given for each tribe.

| **Defined groups** | **Cross-validation** | | | | | | | |
| --- | --- | --- | --- | --- | --- | --- | --- | --- |
|  | Ancylaini | Bombini | Electrapini | Emphorini | Euglossini | Melikertini | Tetrapediini | **%** |
| Ancylaini | **25** | 0 | 0 | 0 | 0 | 0 | 0 | **100** |
| Bombini | 0 | **841** | 0 | 0 | 0 | 0 | 0 | **100** |
| Electrapini | 0 | 0 | **4** | 0 | 0 | **2** | **0** | **66.67** |
| Emphorini | 0 | 0 | 0 | **27** | 0 | 0 | 0 | **100** |
| Euglossini | 0 | 0 | 0 | 0 | **55** | **0** | 0 | **100** |
| Melikertini | 0 | 0 | **1** | 0 | 0 | **2** | 0 | **66.67** |
| Tetrapediini | 0 | 0 | 0 | 0 | 0 | 0 | **16** | **100** |
