# Supplementary material for: Morphometric analysis of fossil bumble bees (Hymenoptera, Apidae, Bombini) reveals their taxonomic affinities
Source: Zookeys. 2019 Nov 21;891:71–118. doi: 10.3897/zookeys.891.36027 (PMC6882928; doi:10.3897/zookeys.891.36027)
Supplement: Supplementary material 7 [file zookeys-891-071-s007.docx]

**Appendix 7 Table S7.** Specimen assignment in subgenera using the cross-validation procedure in the LDA of forewing shape in the third dataset. Original groups are along the rows, predicted groups are along the columns. The hit ratio (HR%) is given for each subgenus.

| **Defined groups** | **Cross-validation** | | | | | | | | | | | | | | | |
| --- | --- | --- | --- | --- | --- | --- | --- | --- | --- | --- | --- | --- | --- | --- | --- | --- |
|  | *Alpigenobombus* | *Alpinobombus* | *Bombias* | *Bombus* s.s. | *Cullumanobombus* | *Kallobombus* | *Megabombus* | *Melanobombus* | *Mendacibombus* | *Orientalibombus* | *Psithyrus* | *Pyrobombus* | *Sibiricobombus* | *Subterraneobombus* | *Thoracobombus* | ***%*** |
| *Alpigenobombus* | **20** | 0 | 0 | 0 | 0 | 0 | **1** | **3** | 0 | 0 | 0 | **1** | 0 | 0 | 0 | **80.00** |
| *Alpinobombus* | 0 | **20** | 0 | 0 | 0 | 0 | 0 | 0 | 0 | 0 | 0 | 0 | 0 | 0 | 0 | **100** |
| *Bombias* | 0 | 0 | **13** | 0 | 0 | 0 | 0 | 0 | 0 | 0 | 0 | 0 | **1** | **1** | 0 | **86.67** |
| *Bombus* s.s. | 0 | 0 | 0 | **22** | 0 | 0 | 0 | 0 | 0 | 0 | 0 | **5** | 0 | 0 | 0 | **81.48** |
| *Cullumanobombus* | **2** | 0 | 0 | 0 | **90** | 0 | 0 | **2** | 0 | 0 | 0 | **3** | 0 | **1** | 0 | **91.84** |
| *Kallobombus* | 0 | 0 | 0 | 0 | 0 | **5** | 0 | 0 | 0 | 0 | 0 | 0 | 0 | 0 | 0 | **100** |
| *Megabombus* | 0 | 0 | 0 | 0 | **2** | 0 | **48** | 0 | 0 | 0 | 0 | **3** | 0 | **4** | **2** | **81.36** |
| *Melanobombus* | **2** | 0 | 0 | 0 | **5** | 0 | 0 | **49** | 0 | 0 | 0 | **10** | **1** | 0 | **1** | **72.06** |
| *Mendacibombus* | 0 | 0 | 0 | 0 | 0 | 0 | 0 | 0 | **19** | 0 | 0 | 0 | 0 | 0 | 0 | **100** |
| *Orientalibombus* | 0 | 0 | 0 | 0 | 0 | 0 | **2** | 0 | 0 | **7** | 0 | **1** | 0 | 0 | 0 | **70.00** |
| *Psithyrus* | 0 | 0 | 0 | 0 | 0 | 0 | 0 | 0 | 0 | 0 | **54** | 0 | 0 | 0 | **1** | **98.18** |
| *Pyrobombus* | **2** | 0 | 0 | **1** | **3** | 0 | **2** | **4** | 0 | **1** | 0 | **159** | 0 | **1** | **6** | **88.83** |
| *Sibiricobombus* | 0 | 0 | 0 | 0 | 0 | 0 | 0 | **3** | 0 | 0 | 0 | **1** | **25** | 0 | 0 | **86.21** |
| *Subterraneobombus* | 0 | **1** | 0 | 0 | 0 | 0 | 0 | 0 | 0 | 0 | 0 | **2** | 0 | **31** | **4** | **81.58** |
| *Thoracobombus* | **2** | 0 | 0 | 0 | 0 | **1** | **5** | **1** | 0 | **3** | 0 | **7** | **1** | **1** | **173** | **89.18** |
