# Supplementary material for: Morphometric analysis of fossil bumble bees (Hymenoptera, Apidae, Bombini) reveals their taxonomic affinities
Source: Zookeys. 2019 Nov 21;891:71–118. doi: 10.3897/zookeys.891.36027 (PMC6882928; doi:10.3897/zookeys.891.36027)
Supplement: Supplementary material 8 [file zookeys-891-071-s008.docx]

**Appendix 8 Table S8.** Specimen assignment in subgenera using the cross-validation procedure in the LDA of forewing shape based on males wing shape. Original groups are along the rows, predicted groups are along the columns. The hit ratio (HR%) is given for each family.

|  | **Bombias** | **Cull** | **Melanobombus** | **Mendacibombus** | **%** |
| --- | --- | --- | --- | --- | --- |
| **Bombias** | 12 | 0 | 0 | 0 | 100 |
| **Cull** | 1 | 17 | 5 | 0 | 73.91 |
| **Melanobombus** | 0 | 5 | 23 | 0 | 82.14 |
| **Mendacibombus** | 0 | 1 | 0 | 18 | 94.74 |
