# Supplementary material for: Morphometric analysis of fossil bumble bees (Hymenoptera, Apidae, Bombini) reveals their taxonomic affinities
Source: Zookeys. 2019 Nov 21;891:71–118. doi: 10.3897/zookeys.891.36027 (PMC6882928; doi:10.3897/zookeys.891.36027)
Supplement: Supplementary material 9 [file zookeys-891-071-s009.docx]

**Appendix 9 Table S9.** Mahalanobis distances (MD) between families centroids and the 988 specimens, and the fossils and families centroids in the first dataset.

|  | **MD individuals - Centroid Min. - Max.** | | ***B. cerdanyensis - Centr. MD (p-value)*** | ***B. pristinus - Centr. MD (p-value)*** | ***B. vetustus - Centr. MD (p-value)*** | ***B. anacolus - Centr. MD (p-value)*** | ***B. dilectus - Centr. MD (p-value)*** | ***B. luianus - Centr. MD (p-value)*** |
| --- | --- | --- | --- | --- | --- | --- | --- | --- |
| **Andrenidae** | 1.091 - 3.940 | | 8.363 (4.073e-14) | 8.668 (2.093e-16) | 11.0462 (1.737e-23) | 10.298 (1.231e-19) | 9.966 (2.042e-18) | 9.441 (7.024e-19) |
| **Apidae** | 0.474 - 5.910 | | **3.346 (1.000)** | **2.417 (1.000)** | **4.486 (1.000)** | **4.678 (1.000)** | **4.558 (1.000)** | **2.906 (1.000)** |
| **Colletidae** | 0.934 - 3.986 | | 7.039 (1.120e-09) | 5.503 (1.179e-06) | 6.556 (2.599e-06) | 8.213 (3.047e-11) | 8.101 (4.353e-11) | 6.933 (5.968e-10) |
| **Halictidae** | 0.940 - 4.912 | | 8.195 (3.284e-13) | 7.455 (7.392e-12) | 9.116 (9.880e-15) | 8.987 (7.679e-14) | 9.433 (7.184e-16) | 8.035 (3.048e-13) |
| **Megachilidae** | 2.190 - 4.039 | | 10.148 (5.016e-22) | 9.733 (2.131e-21**)** | 10.649 (2.377e-22) | 9.377 (1.951e-16) | 9.932 (5.232e-19) | 9.863 (2.192e-21) |
| **Melittidae** | 0.912 - 5.615 | | 6.725 (3.466e-09) | 6.879 (8.403e-11) | 9.062 (2.953e-15) | 7.316 (1.147e-08) | 8.193 (7.321e-12) | 7.235 (2.505e-11) |
| **Stenotritidae** | 1.042 - 4.755 | | 8.106 (5.874e-14) | 7.804 (8.403e-11) | 9.327 (1.217e-16) | 9.698 (8.683e-18) | 10.271 (1.630e-20) | 8.653 (1.522e-16) |
|  |  |  |  |  |  |  |  |  |
|  | **MD individuals - Centroid Min. - Max.** | | ***B. randeckensis - Centr. MD (p-value)*** | ***B. trophionus - Centr. MD (p-value)*** | ***B. beskonakensis - Centr. MD (p-value)*** | ***B. patriciae - Centr. MD (p-value)*** | ***C. florissantensis* UCM - Centr. MD (p-value)** | ***C. florissantensis* MCZ - Centr. MD (p-value)** |
| **Andrenidae** | 1.091 - 3.940 | | 8.766 (8.599e-17) | 8.398 (4.758e-16) | 9.357 (1.531e-17) | 7.789 (1.252e-11) | 10.054 (8.356e-20) | 9.586 (2.732e-19) |
| **Apidae** | 0.474 - 5.910 | | **2.402 (1.000)** | **1.695 (1.000)** | **3.611 (1.000)** | **3.659 (1.000)** | **4.018 (1.000)** | **3.053 (1.000)** |
| **Colletidae** | 0.934 - 3.986 | | 6.184 (2.125e-08) | 5.472 (3.151e-07) | 6.601 (5.617e-08) | 6.313 (4.268e-07) | 7.961 (1.325e-11) | 6.924 (9.841e-10) |
| **Halictidae** | 0.940 - 4.912 | | 7.624 (1.991e-12) | 7.218 (9.560e-12) | 8.834 (3.578e-15) | 8.072 (2.668e-12) | 8.814 (2.026e-14) | 8.580 (5.104e-15) |
| **Megachilidae** | 2.190 - 4.039 | | 9.362 (7.114e-20) | 8.076 (1.234e-15) | 9.601 (2.794e-19) | 9.463 (1.235e-18) | 10.477 (1.998e-22) | 9.381 (3.513e-19) |
| **Melittidae** | 0.912 - 5.615 | | 6.684 (3.036e-10) | 5.347 (2.218e-07) | 6.950 (1.880e-09) | 6.113 (5.277e-07) | 8.281 (3.531e-13) | 7.376 (1.384e-11) |
| **Stenotritidae** | 1.042 - 4.755 | | 8.191 (1.956e-15) | 6.274 (4.818e-10) | 8.668 (1.331e-15) | 8.107 (1.741e-13) | 10.031 (1.831e-20) | 8.938 (1.928e-17) |

|  |  |  |  |  |  |  |  |  |
| --- | --- | --- | --- | --- | --- | --- | --- | --- |
|  | **MD individuals - Centroid Min. - Max.** | | ***O. cuspidatus - Centr. MD (p-value)*** |  |  |  |  |  |
| **Andrenidae** | 1.091 - 3.940 | | 9.791 (9.335e-20) |  |  |  |  |  |
| **Apidae** | 0.474 - 5.910 | | **3.338 (1.000)** |  |  |  |  |  |
| **Colletidae** | 0.934 - 3.986 | | 7.618 (1.571e-11) |  |  |  |  |  |
| **Halictidae** | 0.940 - 4.912 | | 8.581 (1.261e-14) |  |  |  |  |  |
| **Megachilidae** | 2.190 - 4.039 | | 9.079 (1.410e-17) |  |  |  |  |  |
| **Melittidae** | 0.912 - 5.615 | | 6.765 (2.582e-09) |  |  |  |  |  |
| **Stenotritidae** | 1.042 - 4.755 | | 8.337 (8.595e-15) |  |  |  |  |  |
