# Supplementary material for: Morphometric analysis of fossil bumble bees (Hymenoptera, Apidae, Bombini) reveals their taxonomic affinities
Source: Zookeys. 2019 Nov 21;891:71–118. doi: 10.3897/zookeys.891.36027 (PMC6882928; doi:10.3897/zookeys.891.36027)
Supplement: Supplementary material 12 [file zookeys-891-071-s012.docx]

**Appendix 12 Table S12.** Mahalanobis distances (MD) between tribes centroids and the 973 specimens, and the fossils and tribes centroids in the second dataset

| **Tribe** | **MD individuals - Centroid Min. - Max.** | | ***B. cerdanyensis -* Centr. MD (p-value)** | ***B. pristinus -* Centr. MD (p-value)** | ***B. vetustus -* Centr. MD (p-value)** | ***B. anacolus -* Centr. MD (p-value)** | ***B. dilectus -* Centr. MD (p-value)** | ***B. luianus -* Centr. MD (p-value)** |
| --- | --- | --- | --- | --- | --- | --- | --- | --- |
| Ancylaini | 1.104 - 5.306 | | 21.075 (1.111e-97) | 22.575 (2.001e-110) | 14.665 (8.120e-29) | 18.433 (1.197e-65) | 18.997 (1.143e-62) | 21.636 (4.076e-102) |
| Bombini | 0.511 - 6.667 | | **2.169 (1.000)** | **3.391 (1.000)** | **9.630 (1.000)** | **6.915 (1.000)** | **9.091 (1.000)** | **2.866 (1.000)** |
| Electrapini | 2.923 - 8.379 | | 19.402 (1.359e-83) | 16.636 (1.805e-60) | 13.192 (1.585e-20) | 14.166 (4.608e-36) | 13.951 (3.441e-27) | 17.088 (1.691e-64) |
| Emphorini | 1.763 - 5.598 | | 20.369 (2.734e-9) | 21.561 (1.127e-100) | 16.302 (8.633e-40) | 17.782 (1.687e-60) | 19.102 (1.669e-63) | 21.211 (3.927e-98) |
| Euglossini | 1.200 - 4.160 | | 18.720 (5.469e-77) | 19.010 (6.953e-78) | 15.482 (7.950e-34) | 18.304 (2.790e-64) | 16.653 (3.504e-44) | 17.560 (4.402e-67) |
| Melikertini | 3.626 - 7.511 | | 23.533 (2.056e-122) | 20.886 (2.096e-95) | 19.493 (1.515e-65) | 19.049 (1.395e-71) | 16.675 (1.315e-45) | 21.645 (4.034e-103) |
| Tetrapediini | 1.668 - 4.943 | | 17.013 (4.545e-64) | 18.711 (9.120e-76) | 15.145 (6.616e-32) | 16.865 (1.288e-53) | 11.605 (1.555e-13) | 17.490 (7.078e-67) |
|  |  |  |  |  |  |  |  |  |
| **Tribe** | **MD individuals - Centroid Min. - Max.** | | ***B. randeckensis -* Centr. MD (p-value)** | ***B. trophonius -* Centr. MD (p-value)** | ***B. beskonakensis -* Centr. MD (p-value)** | ***B. patriciae -* Centr. MD (p-value)** | ***C. florissantensis* MCZ - Centr. MD (p-value)** | ***C. florissantensis* UCM - Centr. MD (p-value)** |
| Ancylaini | 1.104 - 5.306 | | 20.632 (2.552e-92) | 19.451 (2.476e-79) | 16.768 (1.575e-54) | 19.111 (6.622e-72) | 16.547 (2.614e-34) | 16.315 (3.176e-38) |
| Bombini | 0.511 - 6.667 | | **3.305 (1.000)** | **4.837 (1.000)** | **6.358 (1.000)** | **6.668 (1.000)** | **11.234 (9.947e-01)** | 12.379 (3.523e-12) |
| Electrapini | 2.923 - 8.379 | | 16.456 (2.626e-59) | 14.930 (3.393e-46) | 18.774 (1.252e-70) | 15.587 (5.610e-46) | 11.260 (5.325e-03) | **9.520 (1.000)** |
| Emphorini | 1.763 - 5.598 | | 19.364 (2.856e-81) | 18.057 (6.084e-68) | 17.730 (1.046e-61) | 18.581 (1.562e-67) | 16.357 (6.424e-33) | 16.489 (1.947e-39) |
| Euglossini | 1.200 - 4.160 | | 18.433 (2.544e-73) | 16.533 (3.454e-56) | 16.301 (7.735e-51) | 19.162 (5.502e-72) | 15.909 (1.812e-29) | 15.332 (3.951e-31) |
| Melikertini | 3.626 - 7.511 | | 20.702 (7.214e-94) | 20.446 (7.222e-89) | 23.868 (4.247e-118) | 19.785 (1.600e-78) | 20.091 (2.031e-63) | 18.797 (4.474e-58) |
| Tetrapediini | 1.668 - 4.943 | | 15.902 (8.949e-55) | 15.794 (2.516e-51) | 14.350 (3.541e-38) | 12.556 (8.191e-27) | 13.398 (8.207e-14) | 13.786 (1.111e-21) |
|  |  |  |  |  |  |  |  |  |

| **Tribe** | **MD individuals - Centroid Min. - Max.** | ***O. cuspidatus -* Centr. MD (p-value)** |
| --- | --- | --- |
| Ancylaini | 1.104 - 5.306 | 17.120 (1.546e-46) |
| Bombini | 0.511 - 6.667 | **9.442 (1.000)** |
| Electrapini | 2.923 - 8.379 | 11.046 (5.195e-10) |
| Emphorini | 1.763 - 5.598 | 15.614 (8.386e-36) |
| Euglossini | 1.200 - 4.160 | 15.779 (1.279e-36) |
| Melikertini | 3.626 - 7.511 | 18.268 (2.757e-56) |
| Tetrapediini | 1.668 - 4.943 | 13.048 (7.601e-20) |
