# Supplementary material for: Morphometric analysis of fossil bumble bees (Hymenoptera, Apidae, Bombini) reveals their taxonomic affinities
Source: Zookeys. 2019 Nov 21;891:71–118. doi: 10.3897/zookeys.891.36027 (PMC6882928; doi:10.3897/zookeys.891.36027)
Supplement: Supplementary material 13 [file zookeys-891-071-s013.docx]

**Appendix 13 Table S13.** Mahalanobis distances (MD) between subgenera centroids and the 841 specimens, and the fossils and subgenera centroids in the third dataset.

| **Subgenus** | **MD individuals - Centroid Min. -Max** | | ***B. cerdanyensis* - Centr. MD (p-value)** | ***B. pristinus* - Centr. MD (p-value)** | ***B. vetustus* - Centr. MD (p-value)** |
| --- | --- | --- | --- | --- | --- |
| *Alpigenobombus* | 2.163 - 4.149 | | 5.180 (2.674e-02) | 8.105 (1.887e-06) | 19.454 (1.605e-16) |
| *Alpinobombus* | 2.229 - 5.109 | | 8.359 (9.651e-12) | 9.246 (7.573e-11) | 19.424 (2.307e-16) |
| *Bombias* | 2.435 - 6.057 | | 10.803 (4.870e-22) | 8.865 (1.792e-09) | **17.456 (9.989e-01)** |
| *Bombus* s.s. | 1.343 - 5.800 | | 7.104 (2.125e-07) | 7.240 (1.553e-03) | 19.802 (1.880e-19) |
| *Cullumanobombus* | 2.187 - 7.187 | | 5.846 (2.667e-03) | **6.504 (8.889e-01)** | 19.000 (3.938e-12) |
| *Kallobombus* | 2.408 - 2.822 | | 8.348 (2.632e-12) | 9.251 (1.818e-11) | 22.373 (9.936e-44) |
| *Megabombus* | 2.138 -5.425 | | 5.630 (5.546e-03) | 7.880 (2.703e-05) | 19.977 (1.258e-20) |
| *Melanobombus* | 1.550 - 5.548 | | **4.882 (3.259e-01)** | 6.782 (9.746e-02) | 18.890 (2.158e-11) |
| *Mendacibombus* | 1.898 - 4.239 | | 6.912 (5.767e-07) | 6.947 (8.751e-03) | 17.910 (4.113e-04) |
| *Orientalibombus* | 1.629 - 6.254 | | 5.242 (7.728e-03) | 8.172 (4.369e-07) | 21.034 (8.220e-31) |
| *Psithyrus* | 2.086 - 6.946 | | 6.236 (1.414e-04) | 7.278 (2.405e-03) | 17.944 (6.519e-04) |
| *Pyrobombus* | 1.911 - 6.075 | | 5.158 (2.145e-01) | 7.562 (9.564e-04) | 19.666 (1.813e-17) |
| *Sibiricobombus* | 2.143 - 5.077 | | 6.260 (6.454e-05) | 8.235 (7.582e-07) | 19.691 (1.807e-18) |
| *Subterraneobombus* | 2.170 - 4.961 | | 5.370 (1.492e-02) | 8.022 (5.596e-06) | 19.624 (8.910e-18) |
| *Thoracobombus* | 1.933 - 6.581 | | 5.051 (4.018e-01) | 8.409 (1.190e-06) | 19.830 (7.691e-19) |
|  |  |  |  |  |  |
| **Subgenus** | **MD individuals - Centroid Min. -Max** | | ***B. anacolus* - Centr. MD (p-value)** | ***B. dilectus* - Centr. MD (p-value)** | ***B. luianus* - Centr. MD (p-value)** |
| *Alpigenobombus* | 2.163 - 4.149 | | 11.550 (2.695e-20) | 22.340 (7.881e-34) | 9.204 (2.736e-08) |
| *Alpinobombus* | 2.229 - 5.109 | | 11.803 (1.118e-21) | 23.681 (2.499e-47) | 11.740 (6.457e-20) |
| *Bombias* | 2.435 - 6.057 | | 9.469 (5.078e-11) | **18.590 (1.000)** | 12.562 (2.220e-24) |
| *Bombus* s.s. | 1.343 - 5.800 | | 9.709 (9.152e-12) | 22.261 (4.917e-33) | 8.697 (2.762e-06) |
| *Cullumanobombus* | 2.187 - 7.187 | | 9.004 (2.430e-08) | 20.785 (1.122e-18) | 9.027 (5.372e-07) |
| *Kallobombus* | 2.408 - 2.822 | | 12.301 (6.907e-25) | 23.086 (6.847e-42) | 10.712 (1.646e-15) |
| *Megabombus* | 2.138 -5.425 | | 11.079 (1.304e-17) | 22.819 (3.720e-38) | 9.792 (2.430e-10) |
| *Melanobombus* | 1.550 - 5.548 | | 9.701 (2.487e-11) | 20.797 (6.089e-19) | **7.204 (9.999e-01)** |
| *Mendacibombus* | 1.898 - 4.239 | | **6.537 (1.000)** | **19.322** (1.192e-06) | 8.252 (8.437e-05) |
| *Orientalibombus* | 1.629 - 6.254 | | 11.111 (1.557e-18) | 23.262 (2.326e-43) | 9.523 (5.516e-10) |
| *Psithyrus* | 2.086 - 6.946 | | 11.572 (4.570e-20) | 22.527 (2.615e-35) | 11.630 (6.363e-19) |
| *Pyrobombus* | 1.911 - 6.075 | | 9.796 (2.601e-11) | 22.001 (1.020e-29) | 8.646 (2.852e-05) |
| *Sibiricobombus* | 2.143 - 5.077 | | 9.896 (1.576e-12) | 21.479 (1.411e-25) | 9.490 (2.193e-09) |
| *Subterraneobombus* | 2.170 - 4.961 | | 10.319 (2.864e-14) | 22.231 (1.335e-32) | 9.425 (5.300e-09) |
| *Thoracobombus* | 1.933 - 6.581 | | 11.150 (1.964e-17) | 22.130 (6.459e-31) | 9.669 (2.645e-09) |
|  |  |  |  |  |  |

| **Subgenus** | **MD individuals - Centroid Min. -Max** | | ***B. randeckensis* - Centr. MD (p-value)** | ***B. trophonius* - Centr. MD (p-value)** | ***B. beskonakensis* - Centr. MD (p-value)** |
| --- | --- | --- | --- | --- | --- |
| *Alpigenobombus* | 2.163 - 4.149 | | 6.300 (7.802e-06) | 8.232 (1.253e-06) | 11.167 (4.898e-16) |
| *Alpinobombus* | 2.229 - 5.109 | | 8.415 (1.093e-12) | 10.653 (1.178e-16) | 10.495 (5.664e-13) |
| *Bombias* | 2.435 - 6.057 | | 8.333 (1.630e-12) | 9.540 (6.711e-12) | 12.256 (8.476e-22) |
| *Bombus* s.s. | 1.343 - 5.800 | | 6.513 (2.164e-06) | 7.466 (5.541e-04) | 10.302 (5.727e-12) |
| *Cullumanobombus* | 2.187 - 7.187 | | **4.418 (7.365e-01)** | **6.582 (9.994e-01)** | 9.298 (3.901e-07) |
| *Kallobombus* | 2.408 - 2.822 | | 8.198 (1.654e-12) | 11.582 (9.643e-22) | 13.217 (1.366e-27) |
| *Megabombus* | 2.138 -5.425 | | 6.466 (6.409e-06) | 9.581 (1.783e-11) | 10.003 (2.586e-10) |
| *Melanobombus* | 1.550 - 5.548 | | 4.566 (2.622e-01) | 7.840 (7.988e-05) | 10.093 (1.210e-10) |
| *Mendacibombus* | 1.898 - 4.239 | | 6.102 (2.024e-05) | 9.588 (5.372e-12) | **7.325 (1.000)** |
| *Orientalibombus* | 1.629 - 6.254 | | 7.008 (2.805e-08) | 9.361 (2.444e-11) | 9.613 (2.020e-09) |
| *Psithyrus* | 2.086 - 6.946 | | 7.868 (2.573e-10) | 10.104 (9.678e-14) | 11.530 (1.748e-17) |
| *Pyrobombus* | 1.911 - 6.075 | | 6.097 (1.974e-04) | 8.253 (7.579e-06) | 10.099 (3.004e-10) |
| *Sibiricobombus* | 2.143 - 5.077 | | 6.352 (6.515e-06) | 8.536 (1.137e-07) | 10.068 (6.649e-11) |
| *Subterraneobombus* | 2.170 - 4.961 | | 5.545 (1.041e-03) | 9.182 (4.863e-10) | 10.244 (1.457e-11) |
| *Thoracobombus* | 1.933 - 6.581 | | 7.491 (1.642e-08) | 9.392 (3.546e-10) | 10.781 (2.622e-13) |
|  |  |  |  |  |  |
| **Subgenus** | **MD individuals - Centroid Min. -Max** | | ***B. patriciae*  - Centr. MD (p-value)** | ***C. florissantensis* UCM *-* Centr. MD (p-value)** | ***C. florissantensis* MCZ *-* Centr. MD (p-value)** |
| *Alpigenobombus* | 2.163 - 4.149 | | 10.225 (4.933e-11) | 16.761 (2.099e-24) | 14.402 (5.331e-20) |
| *Alpinobombus* | 2.229 - 5.109 | | 11.317 (3.047e-16) | 18.514 (6.334e-38) | 16.455 (7.511e-34) |
| *Bombias* | 2.435 - 6.057 | | 8.381 (8.291e-04) | **13.072 (1.000)** | **10.847 (1.000)** |
| *Bombus* s.s. | 1.343 - 5.800 | | 10.249 (4.157e-11) | 17.945 (2.703e-33) | 14.356 (1.119e-19) |
| *Cullumanobombus* | 2.187 - 7.187 | | 9.153 (6.237e-06) | 15.643 (6.105e-16) | 12.817 (4.917e-10) |
| *Kallobombus* | 2.408 - 2.822 | | 8.864 (4.295e-06) | 19.328 (3.203e-45) | 17.015 (1.601e-38) |
| *Megabombus* | 2.138 -5.425 | | 10.272 (7.143e-11) | 17.881 (1.852e-32) | 15.061 (7.652e-24) |
| *Melanobombus* | 1.550 - 5.548 | | 9.244 (1.875e-06) | 16.018 (1.112e-18) | 13.752 (1.366e-15) |
| *Mendacibombus* | 1.898 - 4.239 | | **7.519 (9.991e-01)** | 14.450 (7.303e-09) | 11.930 (5.556e-06) |
| *Orientalibombus* | 1.629 - 6.254 | | 11.803 (5.576e-19) | 18.707 (8.652e-40) | 16.051 (2.681e-31) |
| *Psithyrus* | 2.086 - 6.946 | | 10.358 (2.761e-11) | 16.437 (1.005e-21) | 15.315 (1.506e-25) |
| *Pyrobombus* | 1.911 - 6.075 | | 9.571 (2.268e-07) | 17.179 (1.250e-26) | 14.348 (8.321e-19) |
| *Sibiricobombus* | 2.143 - 5.077 | | 8.875 (2.266e-05) | 16.418 (7.274e-22) | 14.290 (3.110e-19) |
| *Subterraneobombus* | 2.170 - 4.961 | | 9.219 (1.318e-06) | 17.294 (3.660e-28) | 14.138 (3.538e-18) |
| *Thoracobombus* | 1.933 - 6.581 | | 10.035 (2.613e-09) | 17.820 (1.829e-31) | 15.244 (1.571e-24) |
|  |  |  |  |  |  |
| **Subgenus** | **MD individuals - Centroid Min. -Max** | | ***O. cuspidatus* - Centr. MD (p-value)** |  |  |
| *Alpigenobombus* | 2.163 - 4.149 | | 12.353 (3.325e-08) |  |  |
| *Alpinobombus* | 2.229 - 5.109 | | 14.874 (3.344e-23) |  |  |
| *Bombias* | 2.435 - 6.057 | | **10.834 (8.949e-01)** |  |  |
| *Bombus* s.s. | 1.343 - 5.800 | | 12.628 (1.168e-09) |  |  |
| *Cullumanobombus* | 2.187 - 7.187 | | 11.202 (1.005e-01) |  |  |
| *Kallobombus* | 2.408 - 2.822 | | 14.578 (6.476e-22) |  |  |
| *Megabombus* | 2.138 -5.425 | | 12.987 (2.569e-11) |  |  |
| *Melanobombus* | 1.550 - 5.548 | | 11.475 (3.166e-03) |  |  |
| *Mendacibombus* | 1.898 - 4.239 | | 11.484 (8.003e-04) |  |  |
| *Orientalibombus* | 1.629 - 6.254 | | 13.173 (3.791e-13) |  |  |
| *Psithyrus* | 2.086 - 6.946 | | 12.695 (1.011e-09) |  |  |
| *Pyrobombus* | 1.911 - 6.075 | | 11.777 (2.493e-04) |  |  |
| *Sibiricobombus* | 2.143 - 5.077 | | 11.583 (3.874e-04) |  |  |
| *Subterraneobombus* | 2.170 - 4.961 | | 12.522 (6.221e-09) |  |  |
| *Thoracobombus* | 1.933 - 6.581 | | 12.577 (1.579e-08) |  |  |
